# The p66^Shc^ Adaptor Protein Controls Oxidative Stress Response in Early Bovine Embryos

**DOI:** 10.1371/journal.pone.0086978

**Published:** 2014-01-24

**Authors:** Dean H. Betts, Nathan T. Bain, Pavneesh Madan

**Affiliations:** 1 Department of Biomedical Sciences, Ontario Veterinary College, University of Guelph, Guelph, Ontario, Canada; 2 Children’s Health Research Institute, Lawson Health Research Institute, London, Ontario, Canada; Cincinnati Children’s Medical Center, United States of America

## Abstract

The *in vitro* production of mammalian embryos suffers from high frequencies of developmental failure due to excessive levels of permanent embryo arrest and apoptosis caused by oxidative stress. The p66Shc stress adaptor protein controls oxidative stress response of somatic cells by regulating intracellular ROS levels through multiple pathways, including mitochondrial ROS generation and the repression of antioxidant gene expression. We have previously demonstrated a strong relationship with elevated p66Shc levels, reduced antioxidant levels and greater intracellular ROS generation with the high incidence of permanent cell cycle arrest of 2–4 cell embryos cultured under high oxygen tensions or after oxidant treatment. The main objective of this study was to establish a functional role for p66Shc in regulating the oxidative stress response during early embryo development. Using RNA interference in bovine zygotes we show that p66Shc knockdown embryos exhibited increased MnSOD levels, reduced intracellular ROS and DNA damage that resulted in a greater propensity for development to the blastocyst stage. P66Shc knockdown embryos were stress resistant exhibiting significantly reduced intracellular ROS levels, DNA damage, permanent 2–4 cell embryo arrest and diminished apoptosis frequencies after oxidant treatment. The results of this study demonstrate that p66Shc controls the oxidative stress response in early mammalian embryos. Small molecule inhibition of p66Shc may be a viable clinical therapy to increase the developmental potential of *in vitro* produced mammalian embryos.

## Introduction

Assisted reproductive technologies (ART) involving the *in vitro* production of preimplantation stage embryos are imperative for the treatment of infertility and/or for fertility management in both human and veterinary reproductive medicine [1], [2]. As of 2012, the number of ART babies born has reached an estimated 5 million since the world’s first, Louise Brown, who was born in July 1978 [3]. Similarily, the total number of *in vitro* produced (IVP) bovine embryos transferred to recipients worldwide in 2011 alone was 373,836 [4]. Despite this widespread use, the fertilization and culturing of early human and livestock embryos in suboptimal, foreign microenvironments leads to a high frequency (50–70%) of early embryonic demise during this 5–7 days of *in vitro* development [5]–[8]. This high incidence of developmental failure has been attributed to a wide range of non mutually exclusive dysfunctions including poor gamete quality [9], chromosomal abnormalities [10], telomere uncapping [11], [12], and suboptimal culture conditions that induce oxidative stress [13], [14].

Numerous studies in many animals including livestock species such as cattle have demonstrated the detrimental effects of culturing gametes and embryos in non-physiological culture systems and are considered valuable models for developing reproductive biotechnologies and evaluating the effects of ART in humans [15]–[19]. *In vitro* culture promotes excessive reactive oxygen species (ROS) production that can override an embryo’s antioxidant defenses producing oxidative stress that triggers apoptosis, necrosis and/or permanent cell cycle arrest in the developing early embryo [6], [12], [20], [21]. Embryos deemed to have a low developmental potential of reaching the blastocyst stage *in vitro* generate significantly elevated levels of intracellular ROS compared to embryos that have a greater tendency for blastocyst formation [22]. Culture regimes utilizing reduced oxygen concentrations [16], [23]–[25] or antioxidant supplementation [24], [26], [27] have improved blastocyst development by reducing intracellular ROS production [28], [29].

We have begun to examine the biochemical and molecular pathway(s) that characterize and control cellular redox state in early embryos. Firstly, the early embryo response to ROS is developmentally regulated [27]. Endogenously generated ROS and/or exogenous ROS treatment elicits a dose-dependent, detrimental effect on early development triggering either permanent embryo arrest (a senescence-like event) or apoptosis depending on the preimplantation stage exposed to the oxidative stress [12], [20], [27], [29]. Conversely, antioxidant enzyme supplementation of the culture medium can limitedly reduce ROS-induced developmental failures [29] suggesting that an optimum redox state is required for proper embryo development. We have identified the stress adaptor protein p66Shc as a major regulator of cellular redox state in early embryos [22]. The reduced developmental potential of embryos is associated with lower levels of antioxidants and elevated quantities of intracellular ROS generation, DNA damage and activated p66Shc [22], [28], [29]. Levels of p66Shc are increased by high atmospheric oxygen or exogenous ROS treatments and are significantly diminished by low oxygen tension or catalase supplementation of the embryo culture media [29]. RNA interference-mediated knockdown of p66Shc in immature bovine oocytes before *in vitro* fertilization (IVF) significantly decreased the incidence of permanent embryo arrest, however embryo development to the blastocyst stage was reduced as well [30]. These results suggest that p66Shc mediates early cleavage arrest but is also important for other later events during the preimplantation period.

P66Shc is a 66-kDa Src collagen homologue (Shc) adaptor protein that is one of three main isoforms encoded by the SHC1 gene (p46Shc, p52Shc, p66Shc) [31]. While p46Shc and p52Shc isoforms link activated receptor tyrosine kinases to the Ras pathway by recruitment of the GRB2/SOS complex [32], [33], p66Shc inhibits Ras signaling in response to epidermal growth factor (EGF) [31]. P66Shc also mediates an inhibitory signaling effect on the extracellular signal-regulated kinase (ERK) pathway that is required for actin cytoskeleton polymerization [34] and normal glucose transport control [33], [35]. The p66Shc isoform is also involved in signal pathways that regulate the cellular response to oxidative stress and life span. Through phosphorylation of Serine 36 in its unique N-terminal collagen homology-2 (CH2) domain, p66Shc acts as a downstream target of the tumor suppressor p53 and is essential for the ability of stress-activated p53 to trigger intracellular ROS generation, cytochrome c release, forkhead inactivation and apoptosis [36]–[39]. P66Shc knockout mice exhibit increased longevity due to their resistance to oxidative stress-induced DNA damage, apoptosis and disease [36], [40]–[42].

Herein this study we examined the mechanistic role of p66Shc in controlling the oxidative stress response of early preimplantation stage embryos. Utilizing RNA interference (RNAi)-mediated knockdown of p66Shc in bovine zygotes, we demonstrate that p66Shc regulates intracellular ROS-induced DNA damage that leads to permanent embryo arrest and apoptosis. P66Shc knockdown embryos are stress resistant exhibiting reduced intracellular ROS levels, diminished DNA damage and reduced incidence of developmental failure leading to significantly increased blastocyst frequencies during *in vitro* embryo development.

## Results

### Optimization of siRNA Delivery into Bovine Zygotes

To optimize the delivery of siRNA molecules into zygotes and to evaluate the effects of microinjection on embryo development, zygotes (sample size (n) = 50/replicate (r), r = 3) were injected with a titration series (10 pL, 50 pL, 75 pL) of injection volumes through the modulation of injection pressure and assessed for cleavage division at 32 hpi (Supplemental Table S1). Constant pressure was maintained throughout these experiments at the lowest possible setting, which was found to maintain equilibrium between excessive backflow and seepage. Increasing injection volume was found to negatively correlate with successful cleavage with the maximal volume resulting in the lowest rate of cleavage (54.5±4.1%) while the minimal volume (representing the lower limit of the FemtoJet™ Injection System) resulted in the highest rate of cleavage (82.1±3.2%). The following regiment of injection settings was established based on the results of this experiment: Injection pressure: 75 hPa; Constant pressure: 15 hPa; Injection time: 0.1 sec; Calculated injection volume: 10 pL. To ensure the effective transfer of fluid at the established regiment of pressure/volume settings, groups (n = 75/r, r = 3) of embryos were injected with a FITC-labeled oligonucleotide probe (Supplemental Table S2). Following the injection protocol, a very high proportion (95.3% ±2.9%) of embryos showed intercellular fluorescence, whereas a liposome-based approached failed (Supplemental Figure S1).

**Figure 1:**
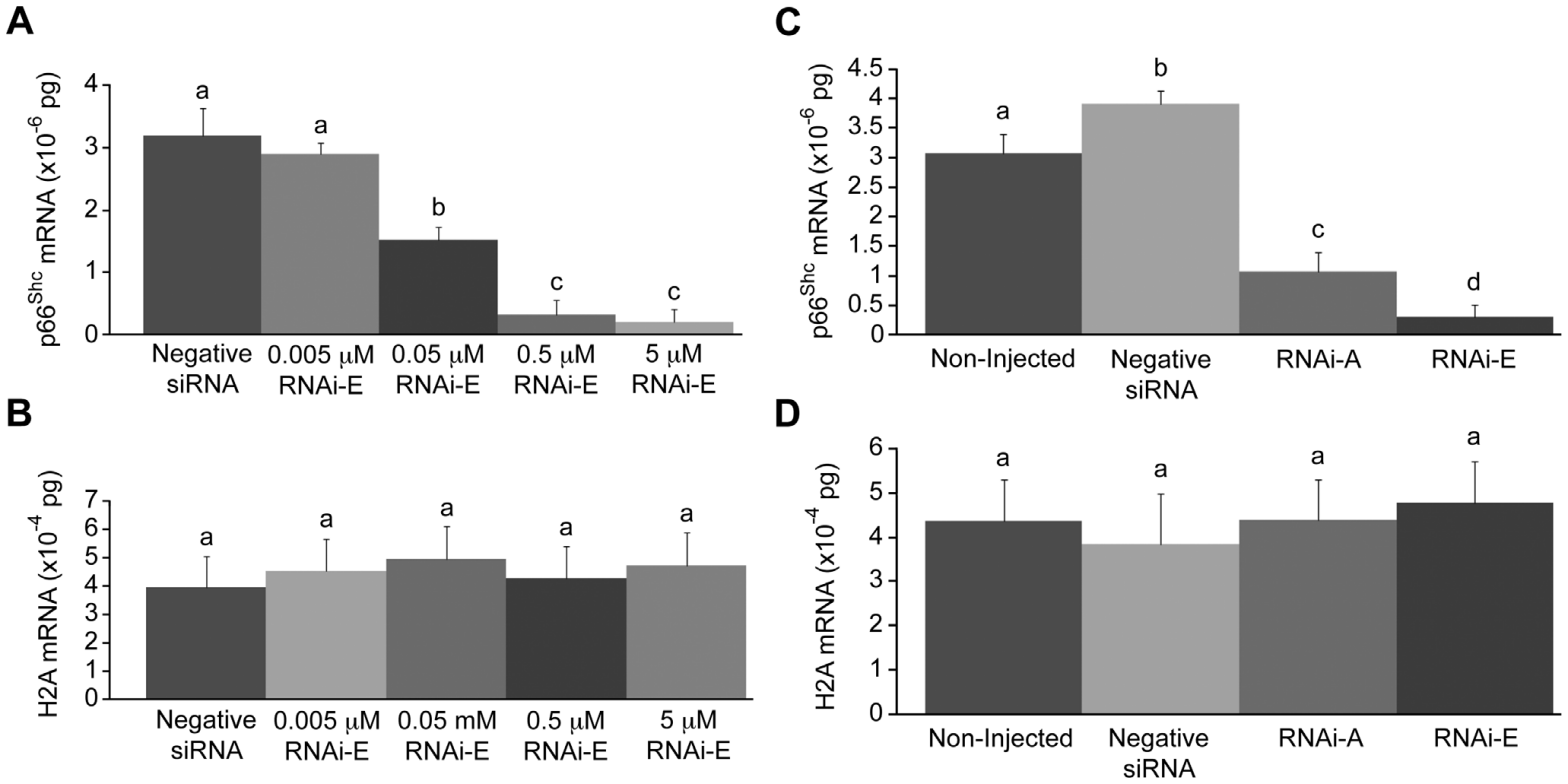
Real-time PCR quantification of p66Shc mRNA levels following RNAi-mediated knockdown of p66Shc in early bovine embryos. Total RNA was extracted from pooled groups of 2–4 cell embryos that, at the zygote stage, were either non-injected, injected with negative control short interfering (si)RNAs, or injected with the p66Shc-specific siRNA molecules (RNAi-A or RNAi-E). (A) Real-time PCR quantification of p66Shc mRNA levels following a titration series of short interfering (si)RNAi-E molecules. The decrease in p66Shc mRNA levels became significant by the 0.05 µM dose level. (B) Histone H2A mRNA was quantified in parallel as an internal control of PCR efficiency. (C) Embryos injected with negative siRNA exhibited significantly higher levels of p66Shc mRNA than non-injected controls. Embryos injected with either RNAi-A or RNAi-E molecules exhibited significantly decreased abundance in p66Shc mRNA. The reduction induced by the RNAi-E molecule was significantly greater than that of the RNAi-A molecule. (D) Histone H2A mRNA was quantified in parallel as a control of PCR efficiency. Different letters above the histogram bars represent significant differences (P<0.05) in transcript abundance. No significant differences in H2A mRNA were noted between groups.

To determine the optimum injection concentration of p66Shc-specific siRNAs, groups (n = 50/r, r = 3) of zygotes were injected with a titration series (non-injected controls, 0.005, 0.05, 0.5, and 5.0 µM) of the p66Shc-specific siRNA molecule RNAi-E and cultured to the 2–4 cell stage (48 hpi) before being pooled and harvested for total RNA and reverse transcription. The 0.005 µM p66Shc siRNA dose did not significantly (P>0.05) reduce p66Shc mRNA (2.90±0.17×10^−6^ pg) compared to embryos injected with negative control siRNA molecules (3.19±0.45×10^−6^ pg) (Figure 1A). The remaining doses each produced significant (P<0.05) decreases in endogenous p66Shc mRNA levels, with the 0.5 and 5.0 µM groups exhibiting the greatest reduction (0.32±0.24×10^−6^ pg; and 0.20±0.21×10^−6^ pg respectively). Overall, compared to p66Shc mRNA levels in control embryos the 0.005 µM siRNA dose resulted in a 9.1% reduction, the 0.05 µM dose resulted in a 53.1% reduction, the 0.5 µM dose resulted in a 90.3% reduction, and the 5.0 µM dose resulted in a 93.8% reduction in p66Shc mRNA abundance. Based on the results of this trial it was determined that the 5.0 µM was the most effective treatment. Histone H2A mRNA, which was quantified in parallel as a control, showed no significant (P>0.05) differences between treatment groups (Figure 1B).

### RNAi-mediated Knockdown of p66Shc in Early Bovine Embryos

To verify the specificity of RNAi-mediated p66Shc knockdown in bovine embryos, groups (n = 50/r, r = 3) of zygotes were injected with two different, non-overlapping siRNA molecules targeting p66Shc mRNA. Zygotes were either non-injected or injected with 10 pL (5.0 µM) of either negative control siRNAs, or one of two p66Shc-specific siRNAs (RNAi-A or RNAi-E). Embryos were cultured to the 2–4 cell stage (32 hpi) before being pooled and processed for total RNA extraction for p66Shc mRNA quantification by Real Time PCR (Figure 1C). Histone H2A mRNA, which was quantified in parallel as siRNA specificity and PCR efficiency controls during this experiment, showed no significant (P>0.05) differences between any experimental groups (Figure 1D). The group of embryos injected with negative siRNA (3.90±0.22×10^−6^ pg) exhibited significantly (P<0.05) higher levels of p66Shc mRNA than non-injected controls (3.07±0.33×10^−6^ pg). Although both anti-p66Shc siRNA molecules produced a significant (P<0.05) decrease in p66Shc mRNA abundance, RNAi-E (0.29±0.21×10^−6^ pg; 92.6% decrease vs. negative siRNA control) generated a significantly greater reduction than RNAi-A (1.07±0.33×10^−6^ pg; 72.6% decrease vs. negative siRNA control). The results from this experiment indicate that while RNAi-A and RNAi-E both effectively knockdown the expression of p66Shc mRNA, RNAi-E was the most effective anti-p66Shc siRNA molecule and was utilized in the majority of the experiments outlined. Initially, we employed both RNAi-A and RNAi-E molecules to ascertain any developmental/physiological differences caused by varying p66Shc knockdown efficiencies, but after observing no significant differences (P>0.05; see below) between the molecules we conducted further experiments with only the more robust p66Shc-specific siRNA molecule (RNAi-E).

**Figure 2:**
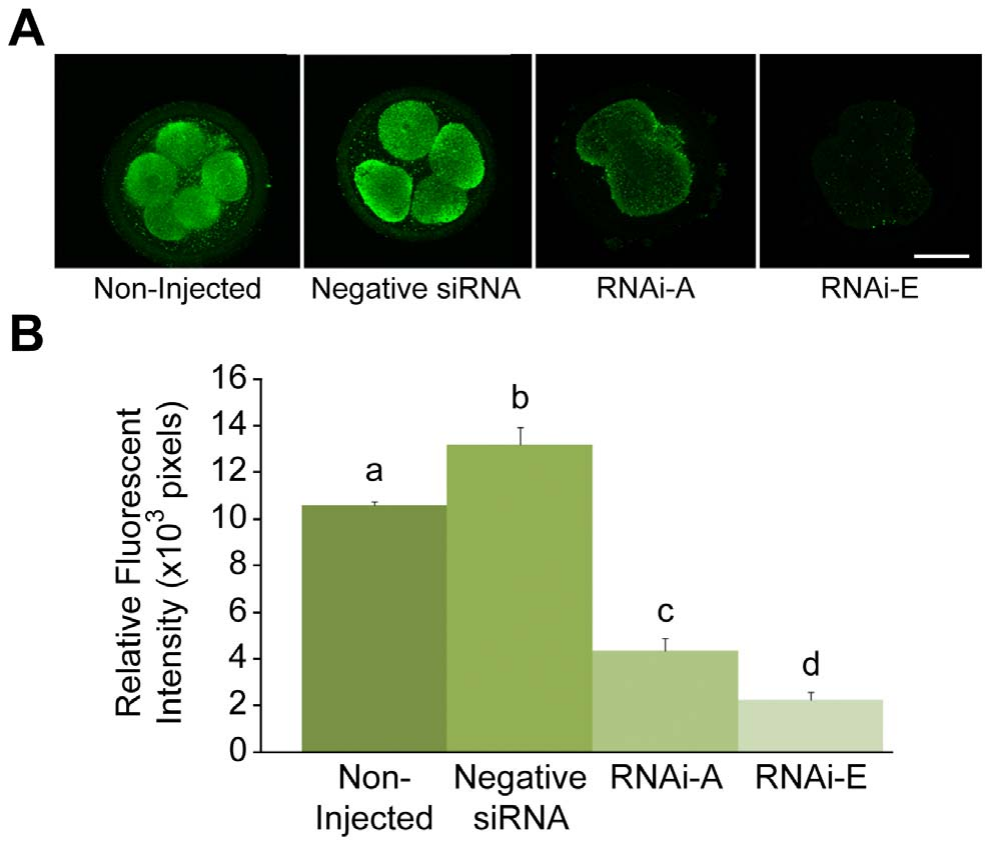
Quantification of total Shc protein levels by semi-quantitative immunofluorescence following RNAi-mediated knockdown of p66Shc in early bovine embryos. Groups of 2–4 cell embryos were immunoassayed for total Sh*c* protein levels by relative immunofluorescent signal intensities. Embryo groups were either non-injected controls; injected with negative control siRNA molecules, or injected with the p66Shc-specific siRNA molecules RNAi-A or RNAi-E. Significant differences (P<0.05) are noted by different letters above the histogram bars. Embryos injected with the negative control siRNA molecule displayed significantly higher levels of Shc protein than non-injected controls. Embryos injected with either RNAi-A or RNAi-E siRNAs exhibited a significant decrease in Shc protein levels. Embryos injected with RNAi-E experienced a significantly greater decrease in Shc protein levels than the RNAi-A injected embryos. Scale bar, 50 µM.

To confirm p66Shc protein levels after p66Shc knockdown, groups (n = 50/r, r = 3) of zygotes were either non-injected or injected with 10 pL (5.0 µM) of either negative siRNAs or p66Shc-specific siRNA molecules RNAi-A or RNAi-E. Following microinjection, embryos were cultured to the 2–4 cell stage (32 hpi) before being processed for whole-mount immunofluorescence for semi-quantitative comparative analysis of total Shc protein (p46Shc, p52Shc and p66Shc isoforms) and activated (serine 36 phosphorylated) p66Shc protein content by relative fluorescent intensities (Figure 2; Supplemental Figure S3). Embryos injected with negative control siRNAs (13.18±0.74×10^3^ pixels) exhibited significantly (P<0.05) higher levels of total Shc protein than non-injected controls (10.61±0.15×10^3^ pixels). While both anti-p66Shc siRNA molecules produced a significant (P<0.05) decrease in total Shc protein, RNAi-E (2.24±0.34×10^3^ pixels; 83.0% decrease vs. negative siRNAs) generated a significantly greater reduction than RNAi-A (4.37±0.52×10^3^ pixels; 66.8% decrease vs. negative siRNAs). In addition, no significant (P>0.05) difference in Serine 36 phosphoryated p66Shc was observed between non-injected embryos (6.54±0.49×10^3^ pixels) and embryos injected with negative siRNA (7.29±0.58×10^3^ pixels). Embryos injected with RNAi-E (0.99±0.39×10^3^ pixels) exhibited levels of phosphorylated-p66Shc protein significantly lower than control groups (Supplemental Figure S3). These results demonstrated efficient and specific knockdown of total and activated p66Shc protein levels, respectively, in early bovine embryos.

To assess the duration of p66Shc knockdown during early bovine development, groups (total n = 50/r, r = 3) of zygotes were injected with 10 pL (5.0 µM) of either negative control siRNAs or p66Shc-specific siRNA molecule RNAi-E and cultured until IVC day 8 (192 hpi). Subgroups (n = 50) were sampled from each group at the 2–4 cell (32 hpi), 9–16 cell (72 hpi), and blastocyst stage (192 hpi). Each subgroup was processed for total RNA extraction for Real Time PCR quantification of p66Shc mRNA levels (Supplemental Figure S2). Histone H2A mRNA was quantified in parallel as a control during this experiment and showed no significant (P>0.05) differences between treatment groups (Supplemental Figure S2). Knockdown of p66Shc mRNA was greatest (P<0.05) at the 2–4 cell stage (negative siRNA = 3.20±0.21×10^−6^ pg; RNAi-E = 0.33±0.25×10^−6^ pg), representing an 89.7% decrease compared to control siRNA injected embryos. By the 9–16 cell stage the difference between the p66Shc and control siRNA group had diminished but remained significantly different (P<0.05; negative siRNA = 2.09±0.27×10^−6^ pg; RNAi-E = 0.78±0.22×10^−6^ pg) at a 62.9% decrease compared to controls. No significant difference (P>0.05) in p66Shc mRNA abundance was observed between the two groups by the blastocyst stage of development.

### p66Shc Knockdown Reduces Early Embryonic Failure

**Figure 3:**
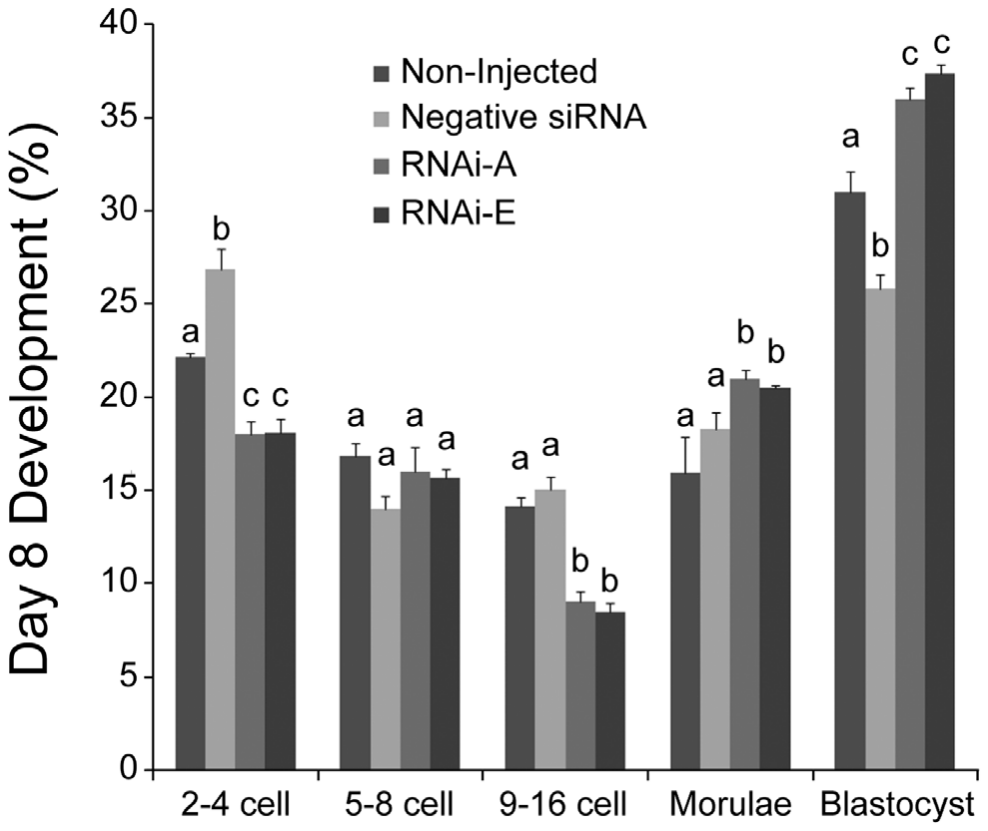
RNAi-mediated knockdown of p66Shc reduces embryo failure and increases blastocyst development. Groups of embryos were either: non-injected, injected with negative control siRNA molecules or injected with the p66Shc-specific siRNA molecules RNAi-A or RNAi-E. The frequency distribution of embryo development was assessed on the 8th day of *in vitro* culture. Different letters above the histogram bars represent significant differences (P<0.05) in developmental frequencies between groups. The proportion of 2–4 cell embryos was increased by the negative control siRNA while both p66Shc specific siRNA molecules produced significant decreases in embryo arrest. The distribution of 5–8 cell embryos was unaffected by any treatment. The proportion of 9–16 cell embryos was significantly decreased in both p66Shc-specific siRNA groups. Negative siRNA injection significantly decreased the proportion of blastocyst stage embryos. The proportion of both morula and blastocyst stage embryos was increased by both of the anti-p66Shc siRNA molecules.

To determine the outcome of RNAi-mediated knockdown of p66Shc on early bovine development, groups (n = 100/r, r = 3) of zygotes were either non-injected, or injected with 10pL (5.0 µM) of negative control siRNAs, RNAi-A, or RNAi-E molecules. Following microinjection, embryos were cultured until IVC day 8 (192 hpi) at which point they were assessed in terms of developmental progress (Figure 3). For this experiment, embryos that had failed to cleave were eliminated from the sample groups after 32 hpi. It should be noted when interpreting these results that a much higher proportion of embryos that underwent microinjection (20–25%) failed to cleave as compared to non-injected controls (8–12%). Non-injected control embryos exhibited the typical “inverted bell” distribution of embryo stages at 192 hpi. Embryos injected with the negative siRNA experienced a significantly (P<0.05) increased frequency of embryo failure at the 2–4 cell and 9–16 cell stage compared to non-injected controls. Additionally, the proportion of blastocysts (25.81±0.23%) was significantly (P<0.05) lower amongst negative siRNA injected embryos than all other groups. Conversely, embryos injected with either p66Shc-specific RNAi-A or RNAi-E siRNAs generated remarkably similar developmental profiles, remaining statistically similar within each developmental stage. Both RNAi-A and RNAi-treated embryos experienced significantly (P<0.05) less developmental failure at the 2–4 cell and 9–16 cell stages. Additionally, the proportion of blastocyst-stage embryos (RNAi-A = 36.0±0.58%; RNAi-E = 37.35±0.50%) was significantly (P<0.05) higher than even the non-injected control (30.97±1.16%) and negative siRNAs injected (25.81±0.77%) embryos. These results indicate that RNAi-mediated knockdown of p66Shc significantly reduces the rate of developmental failure at the 2–4 cell (permanent embryo arrest) and 9–16 cell (embryo apoptosis) stages while increasing the frequency of blastocyst development.

### RNAi-mediated Knockdown of p66Shc Reduces Intracellular ROS Levels

**Figure 4:**
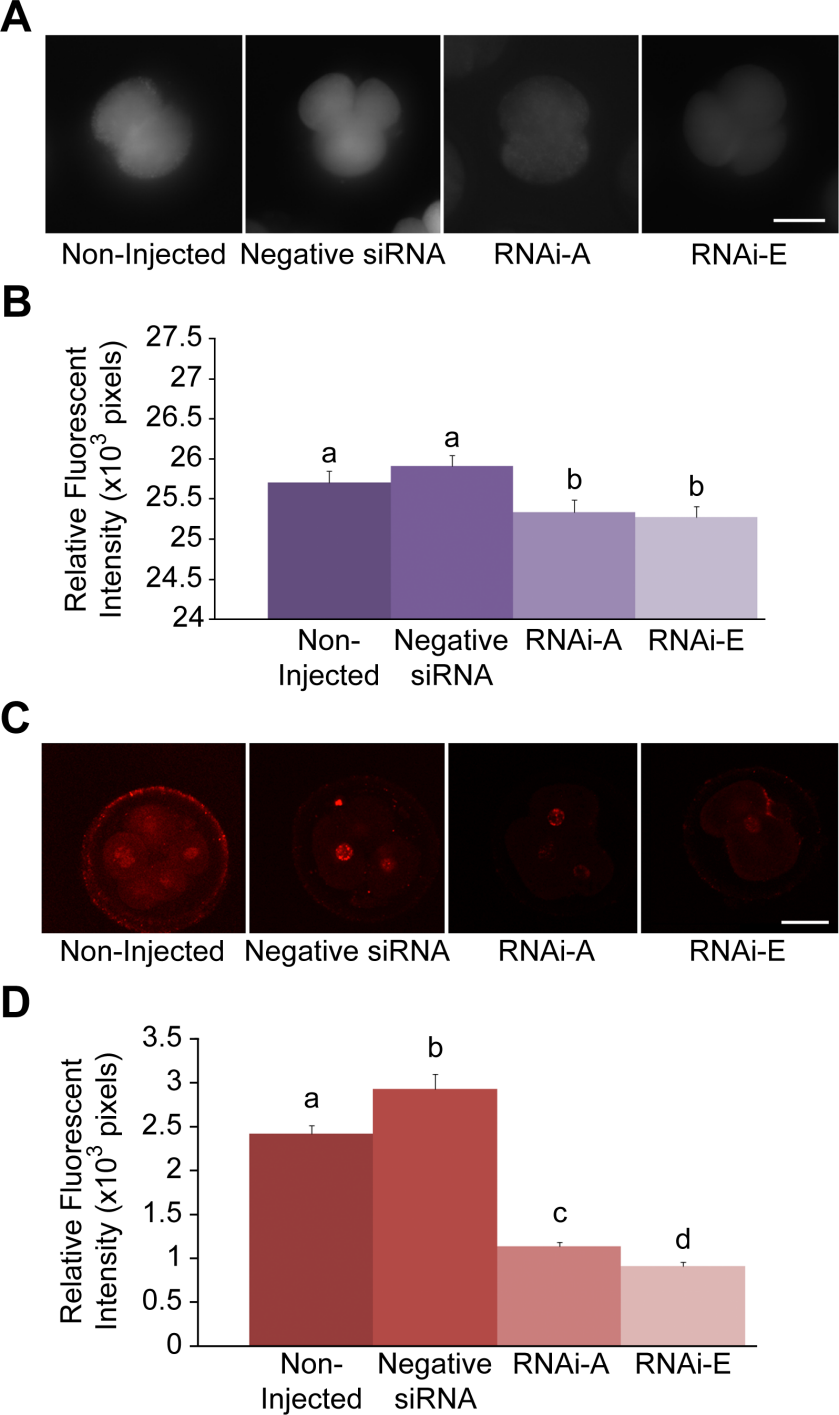
Decreased intracellular ROS and associated DNA damage following RNAi-mediated knockdown of p66Shc in early bovine embryos. Groups of 2–4 cell embryos were stained with 2′,7′-di- chlorofluorescein (DCF) and immunoassayed for phosphorylated γ-H2A.X protein to measure intracellular reactive oxygen species (ROS) content and DNA damage, respectively. Embryos groups were either non-injected controls, injected with negative control siRNA molecules, or injected with the p66Shc-specific siRNA molecules RNAi-A or RNAi-E. (A) Representative fluorescent images of DCF stained embryos. (B) A slight, non-significant increase in intracellular ROS was observed in negative siRNA-injected embryos over non-injected controls. Embryos injected with p66Shc-specific siRNA molecules exhibited significantly (P<0.05) reduced ROS levels. Different letters above the histogram bars represent significant differences (P<0.05) in intracellular ROS intensities. There were no significant difference between RNAi-A and RNAi-E injected embryos observed. (C & D) Quantification of ROS-associated DNA damage following RNAi-mediated p66Shc knockdown. Groups of 2–4 cell embryos were stained for phosphorylated γ-H2A.X protein for quantification by relative immunofluorescent signal intensities. Embryos were: non-injected (controls), injected with negative control siRNA molecule, or injected with the p66Shc-specific siRNA molecules RNAi-A or RNAi-E. (C) Representative fluorescent images of phospho-γ-H2A.X stained embryos. (D) Negative siRNAinjected embryos exhibited significantly higher levels of phosphorylated γ-H2A.X protein than non-injected controls. Embryos injected with either RNAi-A or RNAi-E molecules exhibited a significant decrease in phosphorylated γ-H2A.X signal intensities compared to controls. Different letters above the histogram bars represent significant differences (P<0.05) in mean phospho-γ-H2A.X fluorescent intensities. Scale bar, 50 µM.

To assess the effect of RNA-mediated p66Shc knockdown on embryo intracellular reactive oxygen species (ROS) content, groups (n = 50/r, r = 3) of zygotes were either non-injected (control), or injected with 10 pL (5.0 µM) of negative control siRNAs, RNAi-A, or RNAi-E molecules. Following RNAi microinjection embryos were cultured to the 2–4 cell stage (32 hpi) before being subjected to 2′, 7′-di- chlorofluorescein (DCF) staining for intracellular ROS quantification by comparative analysis of relative fluorescent intensities (Figure 4A,B). Both control groups [non-injected embryos (25.70±0.15×10^3^ pixels) and negative control siRNAs injected embryos (25.91±0.13×10^3^ pixels)] exhibited significantly (P<0.05) higher levels of intracellular ROS than embryos that had been injected with either RNAi-A (25.33±0.16×10^3^ pixels) or RNAi-E (25.28±0.13×10^3^ pixels). It should be noted that intracellular ROS levels were not significantly (P>0.05) higher amongst negative siRNA injected embryos as compared to non-injected controls. This data indicates that RNAi-mediated knockdown of p66Shc is sufficient in generating a significant decrease in embryo intracellular ROS content at 32 hpi.

### Reduced DNA Damage Following RNAi-mediated Knockdown of p66Shc

To measure the effect of RNAi-mediated p66Shc knockdown on ROS-associated DNA damage, groups (n = 50/r, r = 3) of zygotes were either non-injected or injected with 10 pL (5.0 µM) of negative control siRNAs, RNAi-A, or RNAi-E. Following microinjection embryos were cultured to the 2–4 cell stage (32 hpi) before being processed for whole mount immunofluorescence for semi-quantitative comparative analysis of phosphorylated γ-H2A.X protein content by relative fluorescent intensities (Figure 4C,D). Embryos injected with negative siRNAs exhibited significantly (P<0.05) greater levels of phosphorylated γ-H2A.X fluorescent intensities (2.93±0.17×10^3^ pixels) than non-injected controls (2.42±0.10×10^3^ pixels) or either p66Shc RNAi-injected group. Embryos microinjected with anti-p66Shc siRNAs, exhibited a significant (P<0.05) decrease in phosphorylated γ-H2A.X protein staining with RNAi-E producing the greatest reduction (0.91±0.05×10^3^ pixels). This data supports the view that RNAi-mediated knockdown of p66Shc significantly reduces ROS-associated DNA damage in early bovine embryos.

### Stress-induced Elevation in p66Shc Levels are Reduced in Knockdown Embryos

**Figure 5:**
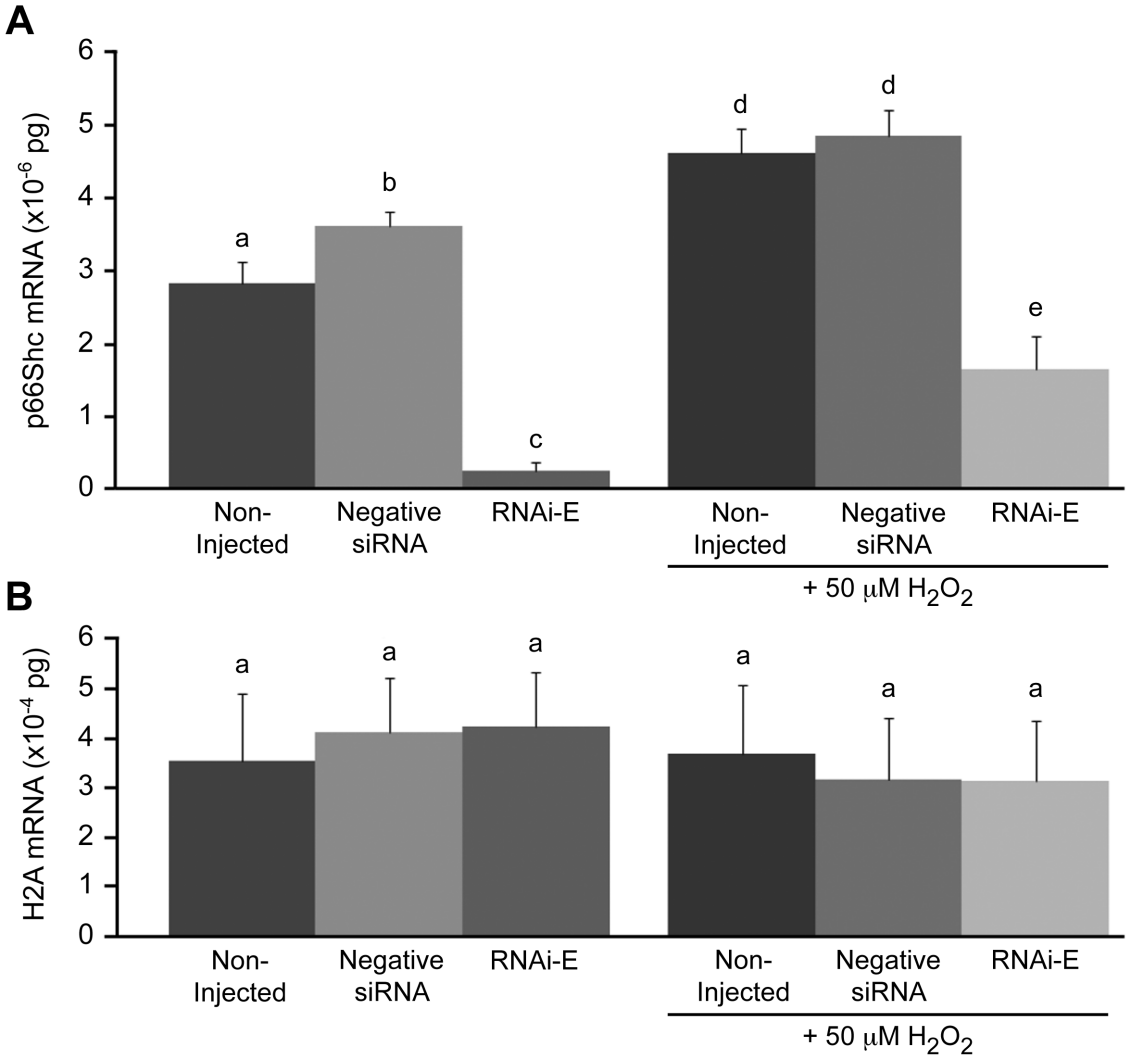
Knockdown of p66Shc ameliorates H_2_O_2_-induced increase in p66Shc mRNA levels in early bovine embryos. Following a combination of siRNA molecule microinjection and hydrogen peroxide (H_2_O_2_; 50 µM) treatments, total RNA was extracted from pooled groups of 5–8 cell embryos for p66Shc mRNA quantification. (A) Non-H_2_O_2_ treated embryos injected with negative siRNA exhibited significantly higher levels of p66Shc mRNA than non-injected controls. Embryos injected with the RNAi-E siRNA molecule exhibited significantly less p66Shc mRNA. H_2_O_2_ treated embryos injected with RNAi-E exhibited significantly lower levels of p66Shc mRNA. However, RNAi-E injected embryos treated with H_2_O_2_ exhibited significantly greater amounts of p66Shc mRNA than RNAi-E injected embryos that were not exposed to H_2_O_2_. (B) Histone H2A mRNA was quantified in parallel as internal controls for PCR efficiency. No significant differences in H2A mRNA were noted between treatment groups. Different letters above the histogram bars represent significant differences (P<0.05) in p66Shc mRNA abundance between groups.

**Figure 6:**
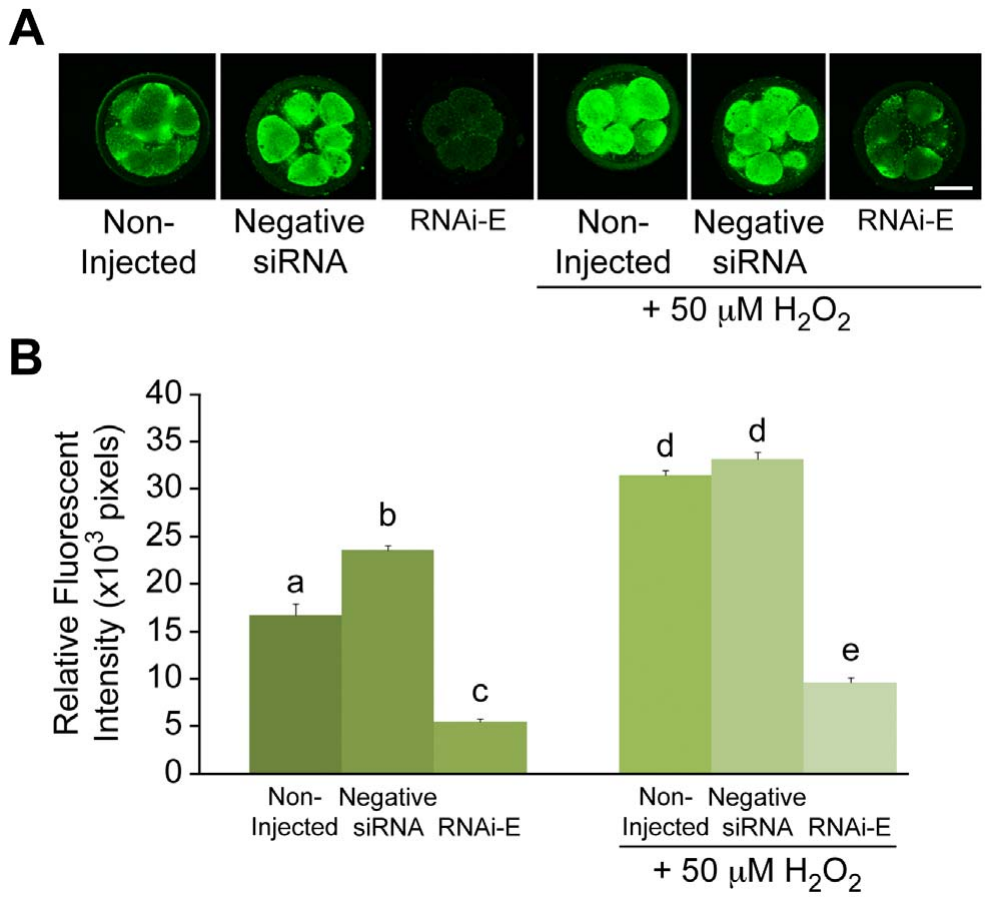
Knockdown of p66Shc ameliorates H_2_O_2_-induced increase in p66Shc protein levels in early bovine embryos. Following a combination of p66Shc siRNA molecule microinjection and H_2_O_2_ (50 µM) treatments, groups of 5–8 cell embryos were immunoassayed for total Shc protein (p46, p52, p66 isoforms) for quantification by relative immunofluorescent signal intensities. Embryos were either: non-injected controls, injected with negative control siRNAs, or injected with p66Shc specific siRNA molecules RNAi-E. (A) Representative images of Shc immunolocalized embryos. (B) Relative Shc fluorescent intensities in Non-H_2_O_2_ treated embryos injected with siRNAs were significantly higher than non-injected controls. Embryos injected with the RNAi-E siRNA molecules displayed significantly reduced Shc immunofluorescent levels. H_2_O_2_ treated embryos injected with RNAi-E siRNA molecule exhibited significantly lower levels of Shc, however, RNAi-E injected embryos treated with H_2_O_2_ exhibited significantly greater amounts of Shc immunofluorescence than similarly injected embryos that were not exposed to H_2_O_2_. Different letters above the histogram bars represent significant differences (P<0.05) in mean fluorescent intensities. Scale bar, 50 µM.

To gauge the effect of oxidative stress on embryo p66Shc levels, groups (n = 100/r, r = 3) of zygotes were either non-injected (control) or injected with 10 pL (5.0 µM) of either negative control siRNAs or with p66Shc-specific siRNA molecules (RNAi-E). Following embryo microinjection and subsequent culture to the 2–4 cell stage (32 hpi) the embryos were subdivided into two subgroups (n = 50), which were either left untreated, or treated with 50 µM hydrogen peroxide (H_2_O_2_) for 1 hr. All subgroups were cultured a further 14 h (48 hpi) before being pooled and processed for total RNA extraction for p66Shc mRNA quantification and whole mount immunofluorescence for semi-quantitative comparative analysis of p66Shc protein content by relative fluorescent intensities (Figures 5 & 6). Histone H2A mRNA was quantified in parallel during these experiments as a control of PCR efficiency (Figure 5B). No significant differences (P>0.05) in H2A mRNA were observed between treatment groups. Embryo subgroups not treated with H_2_O_2_ exhibited the anticipated pattern of p66Shc mRNA abundance with negative siRNA injected embryos displaying significantly (P<0.05) greater levels of p66Shc mRNA (3.61±0.20×10^−6^ pg) than non-injected embryos (2.82±0.30×10^−6^ pg) while RNAi-E injected embryos showed significantly (P<0.05) less (0.24±0.12×10^−6^ pg). Among the subgroup of embryos treated with H_2_O_2_ a similar yet elevated pattern of p66Shc mRNA abundance was observed. Both non-injected (4.62±0.34×10^−6^ pg) and negative siRNA injected (4.85±0.35×10^−6^ pg) embryos exhibited extremely high levels of p66Shc mRNA. As was anticipated, those embryos injected with RNAi-E prior to H_2_O_2_ treatments exhibited significantly (P<0.05) lower quantities of p66Shc mRNA (1.64±0.46×10^−6^ pg) than H_2_O_2_ treated control groups.

Total Shc protein content (Figure 6) strongly resembled the p66Shc mRNA data (above). Non-injected embryo subgroups exhibited the anticipated pattern of total Shc protein abundance with negative siRNA injected embryos exhibiting significantly (P<0.05) greater levels of Shc protein (23.59±0.57×10^3^ pixels) than non-injected embryos (16.73±1.23×10^3^ pixels), while those injected with RNAi-E exhibited significantly (P<0.05) less Shc protein (5.47±0.30×10^3^ pixels). Among the embryo subgroups treated with H_2_O_2_, a similar yet overall increase abundance of p66Shc protein was observed. Both non-injected (31.53±0.49×10^3^ pixels) and negative siRNA injected (33.24±0.67×10^3^ pixels) embryos exhibited extremely high levels (P<0.05) of p66Shc protein (Figure 6). Again, those embryos injected with RNAi-E before H_2_O_2_ treatment exhibited significantly (P<0.05) lower levels of total Shc protein (9.61±0.50×10^3^ pixels) than control groups treated with H_2_O_2_ (Figure 6). The results of these experiment suggest that RNAi-mediated knockdown of p66Shc confers protection against an H_2_O_2_-induced increase in total Shc levels.

### p66Shc Knockdown Embryos Generate Lower Intracellular ROS Levels

**Figure 7:**
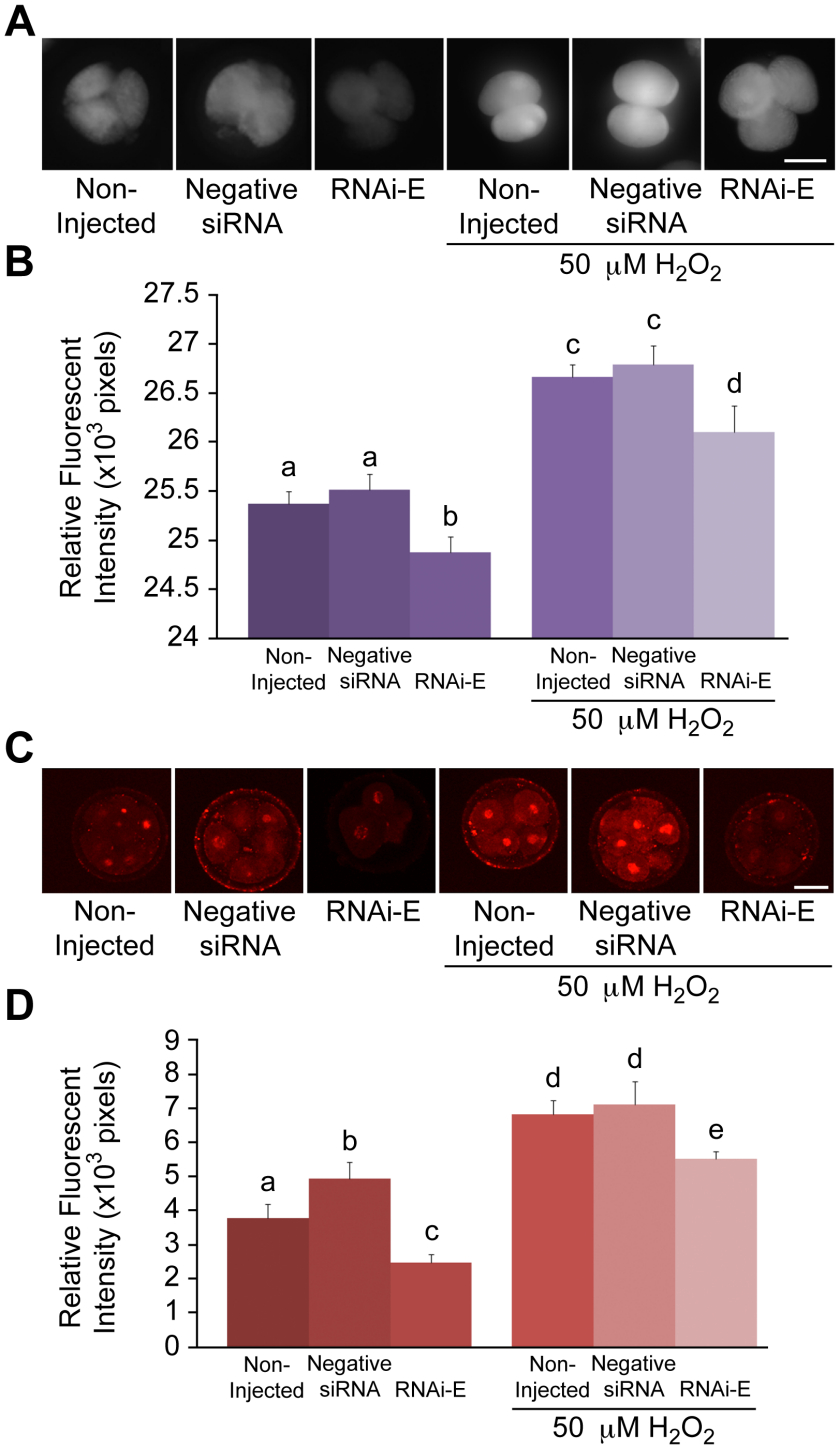
RNAi-mediated knockdown of p66Shc reduces H_2_O_2_-induced ROS accumulation and DNA damage in early bovine embryos. Following a combination of microinjection and H_2_O_2_ (50 µM) treatments, groups of 5–8 cell bovine embryos were stained with 2′,7′-di-chlorofluorescein (DCF) and immunoassayed for phosphorylated γ-H2A.X protein to measure intracellular ROS content and DNA damage, respectively. Embryos were either: non-injected controls, injected with negative control siRNA molecules, or injected with RNAi-E molecules that specifically target p66Shc. (A) Representative fluorescent images of DCF stained embryos. (B) Non-H_2_O_2_ treated embryos injected with RNAi-E exhibited significantly lower levels of intracellular ROS than negative control siRNA-injected and non-injected control embryos. H_2_O_2_ treated embryos injected with RNAi-E exhibited significantly lower ROS levels than negative control siRNA-injected and non-injected controls. However, RNAi-E injected embryos exposed to H_2_O_2_ exhibited significantly greater amounts of intracellular ROS than any groups not exposed to H_2_O_2_. (C) Representative images of phosphorylated γ-H2A.X immunolocalized bovine embryos. (D) Following a combination of siRNA molecule microinjection and H_2_O_2_ treatments, groups of 5–8 cell embryos were stained for phosphorylated γ-H2A.X protein for quantification by relative immunofluorescent signal intensities. Non-H_2_O_2_ treated embryos injected with negative control siRNAs exhibited significantly higher levels of phosphorylated γ-H2A.X protein than non-injected controls. Embryos injected with the RNAi-E siRNA exhibited significantly less phosphorylated γ-H2A.X protein than controls. H_2_O_2_ treatment of RNAi-E-injected embryos resulted in significantly lower levels of phosphorylated γ-H2A.X protein than either control groups. However, RNAi-E injected embryos treated with H_2_O_2_ exhibited significantly greater amounts of γ-H2A.X protein than any groups not exposed to H_2_O_2_. Different letters above the histogram bars represent significant differences (P<0.05) in relative fluorescent intensity between groups. Scale bar, 50 µM.

Groups (n = 100/r, r = 3) of zygotes were either non-injected or injected with 10 pL (5.0 µM) of either negative control siRNAs or p66Shc-specific siRNA RNAi-E. Following microinjection, embryos were cultured to the 2–4 cell stage (32 hpi) and subdivided into two subgroups (n = 50) that were either untreated or treated with 50 µM H_2_O_2_ for 1 hr. Following H_2_O_2_ treatment, embryos were cultured an additional 6 h before being subjected to DCF staining for intracellular ROS quantification by comparative analysis of relative fluorescent intensities (Figure 7A,B). Non-injected (25.37±0.13×10^3^ pixels) and negative siRNA-injected (25.51±0.16×10^3^ pixels) embryos exhibited significantly (P<0.05) higher levels of intracellular ROS than embryos injected with RNAi-E (24.88±0.16×10^3^ pixels). The groups of embryos treated with H_2_O_2_ exhibited a similar, yet overall increased pattern of intracellular ROS levels. Non-injected (26.67±0.19×10^3^ pixels) and negative siRNA-injected (26.79±0.21×10^3^ pixels) embryos that were treated with H_2_O_2_ exhibited significantly (P<0.05) higher levels of ROS than embryos injected with RNAi-E (26.10±0.11×10^3^ pixels). Intracellular ROS content follows a similar correlative pattern as the p66Shc mRNA and Shc protein abundance following the combined effects of p66Shc RNAi and H_2_O_2_ treatment. The reduced intracellular ROS generation following exogenous H_2_O_2_ treatment suggest that p66Shc knockdown by RNAi diminishes the oxidative stress response in early embryos.

### Reduced DNA Damage Following p66Shc Knockdown and H_2_O_2_ Treatment

Since the stress response was found reduced in p66Shc knockdown embryos we wanted to examine their degree of DNA damage. Groups (n = 100/r, r = 3) of zygotes were non-injected or injected with 10 pL (5.0 µM) of either negative control siRNAs or p66Shc-specific RNAi-E. Following microinjection, embryos were cultured to the 2–4 cell stage (32 hpi) and subdivided into two subgroups (n = 50) that were either untreated, or treated with 50 µM H_2_O_2_ for 1 hr. All subgroups were cultured a further 14 h (48 hpi) before being processed for whole mount immunofluorescence for semi-quantitative comparative analysis of the damage marker phosphorylated γ-H2A.X using relative fluorescent intensities (Figure 7C,D). A bimodal pattern of phospho-γ-H2A.X protein abundance was observed within untreated and H_2_O_2_-treated embryo groups. Among the subgroup not treated with H_2_O_2_, embryos injected with negative siRNA (4.93±0.48×10^3^ pixels) exhibited significantly (P<0.05) greater levels of phosphorylated γ-H2A.X protein than non-injected controls (3.78±0.42×10^3^ pixels). Embryos injected with RNAi-E (2.48±0.24×10^3^ pixels) exhibited significantly (P<0.05) less phosphorylated γ-H2A.X protein than either control group. Among the subgroups treated with H_2_O_2_, a slightly different and overall increased pattern of phospho-γ-H2A.X protein abundance was observed. Both non-injected (6.82±0.42×10^3^ pixels) and negative siRNA injected (7.11±0.67×10^3^ pixels) groups exhibited particularly high, statistically similar levels of phosphorylated γ-H2A.X protein content. The group injected with the p66Shc siRNA molecule RNAi-E (5.52±0.21×10^3^ pixels) before H_2_O_2_ treatment exhibited significantly (P<0.05) less phosphorylated γ-H2A.X protein content than H_2_O_2_-treated controls. This data confirms that p66Shc knockdown by RNAi confers protection against ROS-induced DNA damage, even after direct 50 µM H_2_O_2_ treatment. It should be noted that this protective effect, although statistically significant, is only partial since embryos injected with RNAi-E ahead of H_2_O_2_ treatment still exhibited significantly (P<0.05) greater amounts of ROS-associated DNA damage than similarly-injected, H_2_O_2_ untreated embryos.

### p66Shc Knockdown Embryos Exhibit Reduced Frequencies of Stress-induced Permanent Embryo Arrest and Apoptosis

In order to developmentally evaluate the efficacy of this protective effect of p66Shc knockdown against H_2_O_2_-induced DNA damage, groups (n = 100/r, r = 3) of zygotes were either non-injected (control) or microinjected with 10 pL (5.0 µM) of either negative control siRNAs or of p66Shc RNAi molecule RNAi-E. Following microinjection, embryos were cultured to the 2–4 cell stage (32 hpi) before being subjected to a 1 hr treatment of either 100 µM or 50 µM H_2_O_2_. Embryos were cultured an additional 40 h (to 72 hpi) at which point the frequency of permanent 2–4 cell arrest was assessed (Table 1). Embryos treated with 100 µM H_2_O_2_ succumbed to permanent embryo arrest regardless of injection treatment. However, embryos treated with 50 µM H_2_O_2_ exhibited a more complex pattern of embryo arrest. Those injected with negative siRNA before 50 µM H_2_O_2_ treatment exhibited a significantly (P<0.05) higher rate of 2–4 cell arrest than non-injected controls. Most importantly, embryos injected with p66Shc siRNA molecule RNAi-E before the 50 µM H_2_O_2_ treatment exhibited a significantly reduced rate of 2–4 cell arrest compared to both control groups. This data indicates that although certain threshold dose levels of exogenous H_2_O_2_ may be insurmountable, the protective effect of p66Shc RNAi is sufficient to significantly reduce the incidence of stress-induced permanent 2–4 cell embryo arrest.

**Table 1:** The effect of p66Shc knockdown on H_2_O_2_-induced permanent embryo arrest*.

Groups	Untreated	Negative siRNA	RNAi-E
100 µM H_2_O_2_ ¥	98.7±1.3%^a^	100±0%^a^	100±0%^a^
50 µM H_2_O_2_ ¥	40.9±2.9%^a^	69.2±5.5%^b^	32.7±1.8%^c^

¥ n = 100/replicate, 3 replicates.

* Superscript letters denote significant differences (P<0.05) within groups.

**Figure 8:**
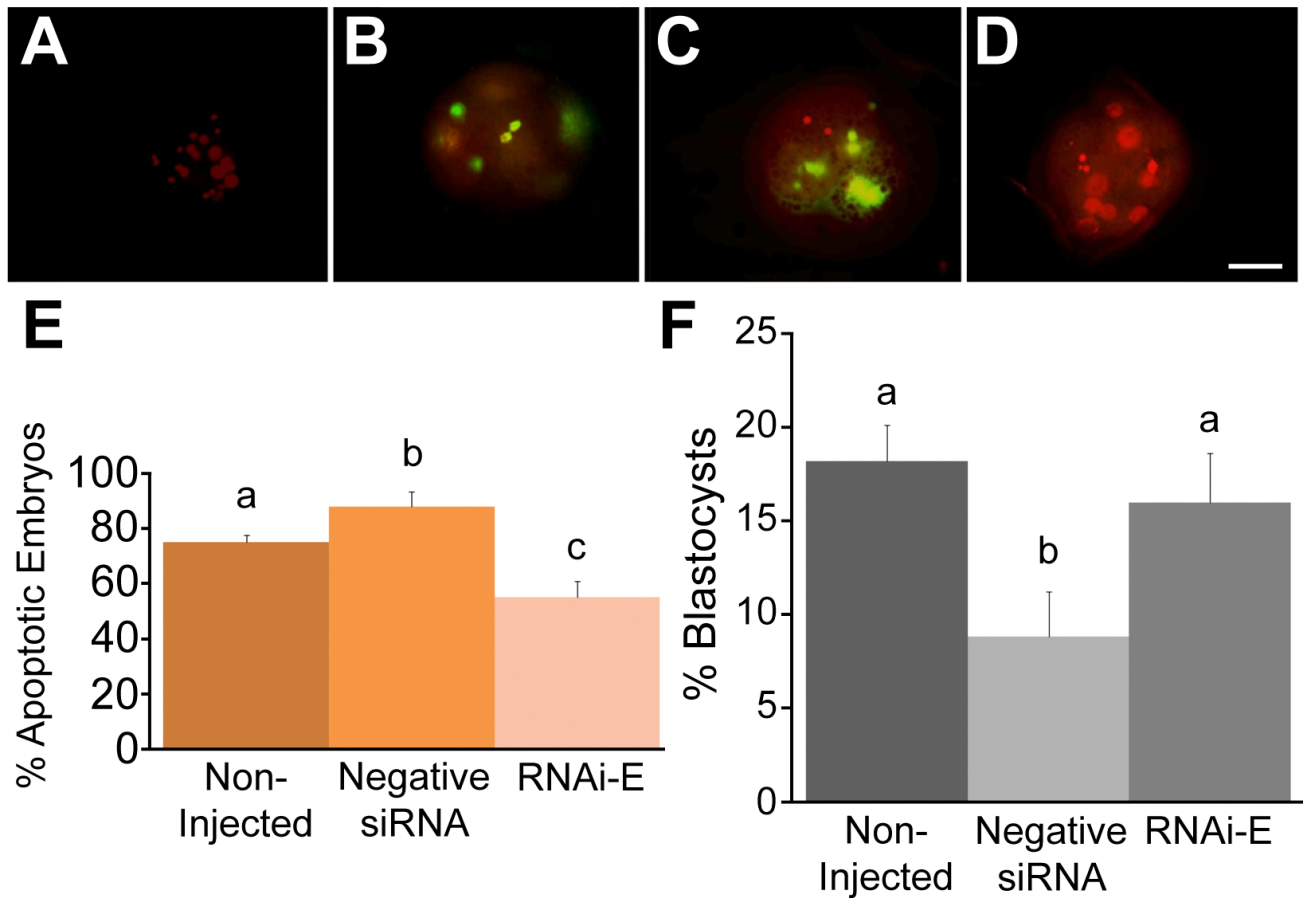
Knockdown of p66Shc ameliorates H_2_O_2_-induced apoptosis and developmental arrest of early bovine embryos. Groups of zygotes (n = 50/r, r = 3) were either: non-injected controls, injected with negative control siRNAs, or injected with p66Shc siRNA molecules (RNAi-E). At the 9–16 cell stage, all groups were subjected to 50 µM H_2_O_2_ treatment for 1 hr. After an overnight incubation, embryos were fixed and stained with a mixture of TUNEL labeling stain (green) and propidium iodide (red) to assess their apoptotic status. (A) Representative negative control embryo stained in the absence of active TUNEL enzyme. (B) Representative positive control embryo subjected to DNase treatment before TUNEL stain. (C) Representative apoptotic 9–16 cell embryo displaying TUNEL positive stain in conjunction with nuclear condensation and fragmentation. (D) Representative non-apoptotic 9–16 cell embryo displaying TUNEL negative staining in conjunction with intact nuclear staining. (E) Summary of the incidence of apoptosis following RNAi microinjection and H_2_O_2_ treatments. While embryos injected with negative siRNA molecules exhibited a greater proportion of apoptosis, embryos injected with RNAi-E exhibited a significantly reduced incidence of apoptosis. (F) The effect of p66Shc knockdown on the frequency of blastocyst formation following H_2_O_2_ treatment. Groups of embryos (non-injected, negative control siRNA- injected, or RNAi-E injected, were subjected to a 1 hr treatment of 50 µM H_2_O_2_ at the 2–4 cell stage after which they were allowed to develop until the 8th day of culture (192 hpi) at which point the frequency of blastocyst development was assessed. A significantly greater proportion of embryos injected with the p66Shc-specific siRNA molecules (RNAi-E) successfully developed into blastocysts compared to those injected with a negative control siRNA. The proportion of blastocysts amongst the RNAi-E group was comparable to that of the non-injected control group. Different letters above the histogram bars represent significant differences (p<0.05). Scale bar, 50 µM.

In order to assess the protective effect of p66Shc RNAi against H_2_O_2_-induced embryo apoptosis, groups (n = 100/r, r = 3) of zygotes were either non-injected or injected with 10 pL (5.0 µM) of either negative control siRNAs or of p66Shc siRNA molecules (RNAi-E). Following microinjection, embryos were cultured to the 9–16 cell stage (72 hpi) and subjected to a 1 hr treatment of 50 µM H_2_O_2_. Embryos were cultured an additional 6 h before their apoptotic status was evaluated by a combination of TUNEL and propidium iodide (PI) staining (Figure 8A–E). An embryo was considered apoptotic only if it displayed both TUNEL-positive staining and nuclear condensation/fragmentation under PI staining in the same blastomere. Embryos injected with negative siRNAs (88.1±5.5%) exhibited a significantly (P<0.05) higher incidence of apoptosis than non-injected embryos (74.8±2.8%). Importantly, embryos injected with RNAi-E (54.5±5.8%) exhibited a significantly (P<0.05) lower incidence of apoptosis than both control groups. This data confirms that the protective effect conferred by p66Shc RNAi against 2–4 cell embryo arrest is also present at the 9–16 cell stage providing protection against H_2_O_2_-induced apoptosis.

RNAi-mediated knockdown of p66Shc in early embryos has demonstrated a protective effect against two forms of stress-induced developmental failure: permanent embryo arrest and embryo apoptosis. As a follow-up to these experiments, the ability of p66Shc knockdown to promote stress resistant development was assessed. Groups (n = 50/r, r = 3) of zygotes were non-injected or microinjected with 10 pL (5.0 µM) of either negative siRNAs or p66Shc RNAi molecules (RNAi-E). Following microinjection, each group of zygote-stage embryos was immediately subjected to a 1 hr treatment of 50 µM H_2_O_2_. Embryos were cultured for 8 days (192 hpi) at which point the frequency of successful blastocyst formation was visually assessed (Figure 8F). Significantly fewer (P<0.05) embryos injected with negative control siRNAs successfully developed into blastocysts after H_2_O_2_ treatment (8.97±2.41%) than non-injected control embryos (18.18±1.94%). A significantly greater (P<0.05) proportion of embryos that were injected with the p66Shc-specific siRNA molecule RNAi-E (15.79±2.66%) successfully developed into blastocysts compared to negative siRNA-injected embryos. However, RNAi-E injected embryos did not successfully develop into blastocysts significantly (P>0.05) more often than non-injected control embryos. Taking into consideration the negative effects of the microinjection process itself on embryo development, this data confirms that p66Shc RNAi does improve the frequency of successful blastocyst development following H_2_O_2_ treatment of zygotes.

### Nuclear Exclusion of FOXO3a is Reduced by p66Shc Knockdown

FOXO3a, a member of the forkhead family of transcription factors, regulates several important antioxidant enzymes. Ser36-phosphorylated p66Shc, mediated by Akt, excludes FOXO3a from the nucleus impairing antioxidant expression [37]. Groups (n = 50/r, r = 3) of embryos were cultured under 5% O_2_ (controls), 20% O_2_, divided by the time of first cleavage division (Early <28 hpi; Late >28 hpi), or subjected to a 1 hr treatment with 50 µM H_2_O_2_ at the 2–4 cell stage (32 hpi). Embryos were cultured to 38 hpi and processed for whole mount immunofluorescence for the localization of FOXO3a protein by confocal microscopy. Embryos were co-stained with DAPI and FOXO3a in order to identify both nuclear boundaries as well as FOXO3a localization. The FOXO3a distribution pattern of each embryo was evaluated individually (Supplementary Figure S4). Embryos exhibiting the “nuclear exclusion” pattern of FOXO3a staining were enumerated (Supplementary Table S3). Nuclear exclusion of FOXO3a was observed to be not significantly different and at a low incidence (4–5%) among all treatment groups with the exception of embryos treated with 50 µM H_2_O_2_, which exhibited significantly (P<0.05) elevated levels (∼55%) of nuclear exclusion.

In addition, groups (n = 50/r, r = 3) of zygote-stage embryos were either non-injected (controls), or injected with 10 pL (5.0 µM) of either negative siRNA molecules or p66Shc-specific siRNAs (RNAi-E). Following microinjection, embryos were cultured to the 2–4 cell stage (38 hpi) before being processed for whole mount immunofluorescence for localization of FOXO3a protein under immunofluorescent confocal microscopy. Embryos co-stained with DAPI and FOXO3a were individually assessed (Supplementary Figure S4). Embryos exhibiting “nuclear exclusion” of FOXO3a immunolocalization were counted (Table 2). A significantly greater (P<0.05) proportion of embryos injected with negative control siRNAs exhibited nuclear exclusion of FOXO3a compared to non-injected controls. Embryos injected with RNAi-E exhibited significantly less (P<0.05) FOXO3a nuclear localization than embryos injected with negative siRNA although nuclear incidence remained significantly above those of non-injected controls. This data indicates FOXO3a translocation can be significantly affected by RNAi-mediated knockdown of p66Shc.

**Table 2:** The incidence of FOXO3a nuclear exclusion following RNAi-mediated knockdown of p66Shc.

Treatment^¥^	% Nuclei FOXO3a Excluded*
Non-Injected	5.4±0.9^a^
Negative control siRNAs	15.1±2.4^b^
p66Shc siRNAs (RNAi-E)	10.2±1.9^c^

• n = 50/replicate, 3 replicates;

* Significant differences (P<0.05) are denoted by superscript letters.

### Elevated MnSOD Activity Following RNAi-mediated Knockdown of p66Shc

**Figure 9:**
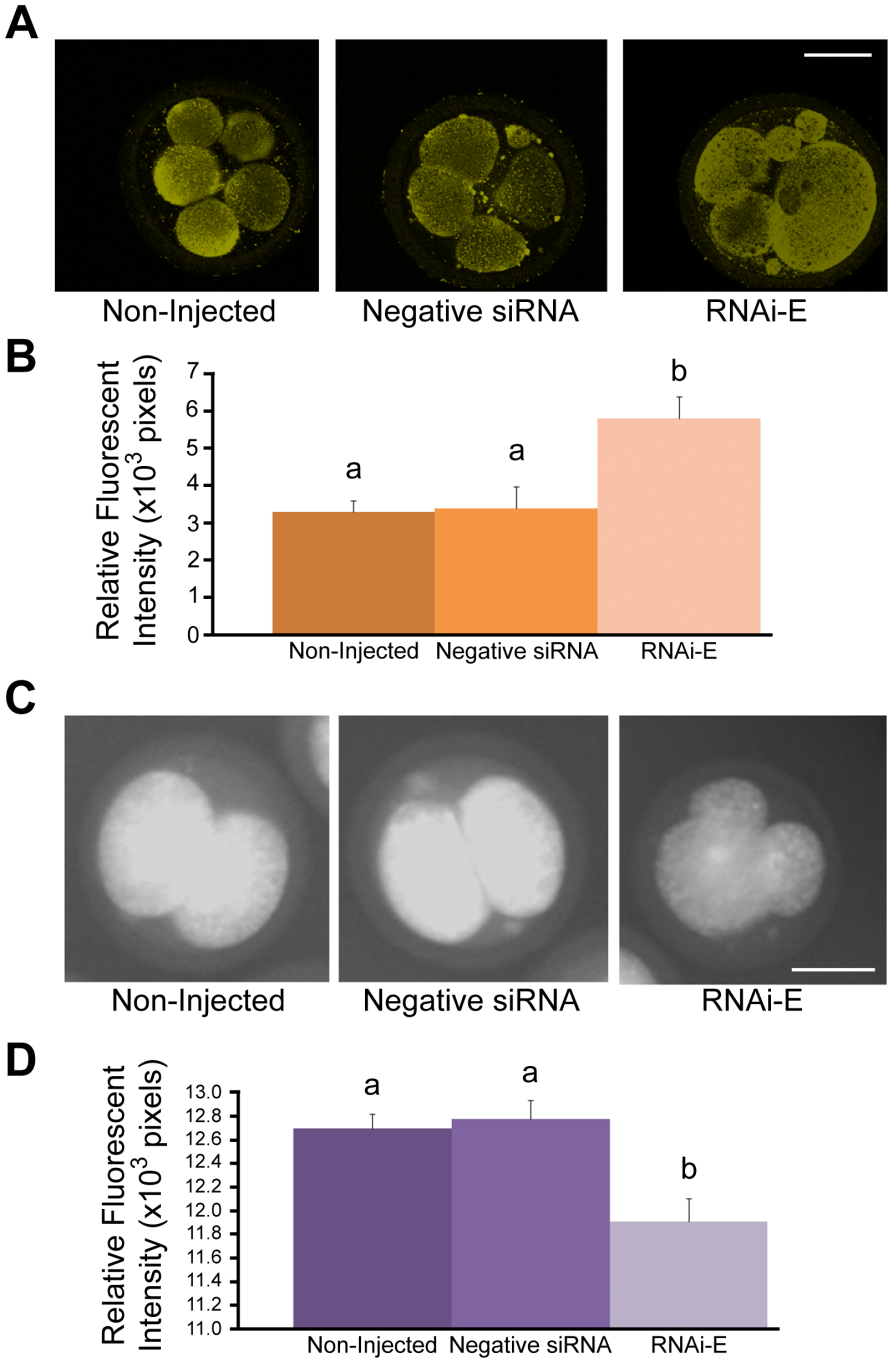
RNAi-mediated knockdown of p66Shc increases MnSOD levels and reduces superoxide production in early bovine embryos. Bovine zygotes were either non-injected (controls), injected with negative control siRNA molecules, or injected with p66Shc-specific siRNAs (RNAi-E). Groups of embryos were either stained for MitoSOX™ detection of superoxide levels or immunostained for MnSOD protein content for quantification by relative fluorescent signal intensities. (A) Representative confocal images of 5–8 cell bovine embryos immunostained for MnSOD protein. (B) Embryos injected with p66Shc siRNA molecule RNAi-E exhibited significantly higher levels of MnSOD protein than control groups. (C) Representative confocal images of 2–4 cell bovine embryos were subjected to MitoSOX™ staining for superoxide quantification by relative fluorescent signal intensity. (D) Embryos injected with p66Shc RNAi molecules exhibited significantly lower levels of superoxide content than control groups. Different letters above the histogram bars represent significant differences (P<0.05) in mean fluorescent intensities. Scale bar, 50 µM.

Groups (n = 50/r, r = 3) of zygotes were either non-injected (controls), or injected with 10 pL (5.0 µM) of either negative control siRNAs or p66Shc-specific RNAi-E molecules. Following microinjection, embryos were cultured to the 5–8 cell stage (48 hpi) before being pooled and harvested for total RNA for the Real Time PCR quantification of catalase and MnSOD mRNA (Supplemental Figure S5), whole mount immunofluorescence for semi-quantitative comparative analysis of catalase and MnSOD protein content by relative fluorescent intensities (Figure 9A,B). No significant differences (P>0.05) in catalase mRNA content were observed between any of the treatment groups, while p66Shc knockdown induced significantly higher (P<0.05) levels of MnSOD mRNA (5.95±0.13×10^−5^ pg) than control groups (non-injected embryos: 5.51±0.14×10^−5^ pg; negative siRNA injected: 5.56±0.10×10^−5^ pg). No significant (P>0.05) differences in H2A mRNA were observed between any treatment groups. Likewise, no significant (P>0.05) differences in catalase protein content were observed between any of the treatment groups (Supplementary Figure S6). However, consistent with MnSOD mRNA data, embryos injected with the p66Shc siRNA molecule RNAi-E (5.80±0.59×10^3^ pixels) exhibited significantly higher (P<0.05) levels of MnSOD protein than either non-injected (3.29±0.29×10^3^ pixels) or negative control siRNA-injected (3.37±0.59×10^3^ pixels) embryos (Figure 9A,B).

To examine the impact of MnSOD upregulation, 2–4 cell embryos (38 hpi; n = 50/r, r = 3) were subjected to live-cell MitoSOX™ staining for comparative superoxide quantification between treatment groups (Figure 9C,D). Embryos injected with RNAi-E (11.91±0.19×10^3^ pixels) exhibited significantly lower (P<0.05) superoxide levels than non-injected (12.70±0.12×10^3^ pixels) and negative control siRNA injected embryos (12.78±0.15×10^3^ pixels). These observations further support the model that RNAi-mediated knockdown of p66Shc confers a protective effect over ROS-associated DNA damage and embryonic failure by positively influencing the expression and activity of at least one key antioxidant enzyme.

## Discussion

The aim of this study was to elucidate the functional role(s) of p66Shc throughout early embryo development. We have previously determined that elevated p66Shc mRNA and protein abundance in early embryos are associated with reduced developmental potential [22], [29]. We have also shown that the early embryo’s response to reactive oxygen species (ROS) is developmental regulated [27] and that activated p66Shc is linked to increased intracellular ROS levels, reduced antioxidant expression and to permanent embryo arrest [22], [28], [29]. Previous microinjections of short hairpin (sh)RNAi molecules targeting p66Shc in immature bovine oocytes successfully knockdown p66Shc and alleviated the 2–4 cell block observed after fertilization, but also resulted in reduced blastocyst development suggesting a role for p66Shc beyond early cleavage arrest [30]. Herein, we mechanistically demonstrate for the first time that RNAi-mediated knockdown of p66Shc at the zygote stage confers oxidative stress resistance throughout early bovine embryo development. P66Shc knockdown embryos are more resistant to oxidant treatment due to increased levels of MnSOD and a reduction in intracellular ROS and DNA damage content that diminishes the frequencies of both permanent embryo arrest and apoptosis resulting in improved blastocyst development *in vitro*.

According to a series of criteria [43], two non-overlapping siRNA sequences (RNA-A, RNA-E) were designed to specifically target the unique CH2 domain of bovine p66Shc. These siRNAs were different than the previous p66Shc shRNA sequence utilized in our previous study [30]. The use of multiple target sequences of the same RNA molecule is an effective means of verifying a genuine loss-of-function effect [44]. While both siRNA molecules induced a significant decrease in p66Shc mRNA and protein, RNAi-E was the more effective of the two. The accessibility to the target sequence may be greater for RNA-E molecules than for RNAi-A siRNAs. A difference in the internal stability of the two siRNA molecules could manifest as different rates of strand selection and entry into the RNA-induced silencing complex (RISC) leading to differential knockdown efficiencies [45], [46]. A significant increase in p66Shc mRNA and protein was observed in embryos injected with negative control siRNAs compared to non-injected embryos. The microinjection process, which involves prolonged exposure to ambient temperature and oxygen tensions, as well as disruption of the cell membrane and components of the cytoskeleton, is a significant stress upon the early bovine embryo [47]. This upregulation may mask the true knockdown effect of p66Shc siRNA molecules, which may prove more effective if introduced in a less traumatic manner. Attempts introducing siRNAs into bovine zygotes using lipofectamine were futile.

A greater proportion of p66Shc knockdown embryos significantly reached the morula and blastocyst stages than control groups following eight days of embryo culture. As the microinjection process itself significantly increased the incidence of embryos that failed to cleave, only those embryos that had undergone at least one successful cleavage division were evaluated. Previous reports have observed a similar decrease in cleavage and blastocyst frequencies after microinjection of bovine oocytes [30] and zygotes [48]. While this strategy suppresses the negative consequences of microinjection, it emphasizes the extent of any RNAi-mediated knockdown effects on development. As a result, we observed a significant decrease in the incidence of embryonic failure at the 2–4 cell and 9–16 cell stages with a concomitant increase in the number of morulae and blasocysts at day eight of embryo culture. A similar reduction in early cleavage arrest was observed in our previous study in which p66Shc was knockdown by shRNA injection of immature bovine oocytes, however blastocyst frequencies were significantly reduced [30]. This decrease in blastocyst production was possibly due to the knockdown of additional Shc isoforms (p52 and p46), which are involved in mitogenic signalling [31] and have been found to be necessary for proper embryo development [49], or more likely due to the re-exppresion of p66Shc at later cleavage stages due to shRNA dilution. In this current study, the knockdown of p66Shc mRNA was greatest amongst 2–4 cell embryos, yet remained significantly different at the 9–16 cell stage. In both studies [30], p66Shc mRNA levels were virtually identical at the blastocyst stage in p66Shc knockdown embryos compared to embryos injected with negative control RNAi molecules suggesting an optimal low p66Shc expression level for developmental competence. While we didn’t evaluate p66Shc levels at the 9–16 cell stage in the previous study we speculate that an earlier introduction of p66Shc RNAi molecules in immature oocytes, although effective in knocking down maternally-derived p66Shc transcripts and alleviating embryo arrest, were diluted out and ineffective of curtailing p66Shc-mediated apoptosis at later cleavage divisions. Alternatively, the negative impact of microinjecting immature oocytes may be more severe than the microinjection of zygotes because of the potential loss of surrounding cumulus cells [50], [51] or that known effects of p66Shc deficiency on altered metabolism [52] and/or glucose uptake [35] were detrimental for further development when p66Shc was knockdown earlier. Although there was a significantly greater degree of p66Shc knockdown by RNAi-E siRNAs compared to RNAi-A molecules, interestingly, their effects on embryo development were very similar. For example, both p66Shc-specific RNAi-A and RNAi-E molecules induced a similar significant reduction in intracellular ROS levels and DNA damage that was not different between the two siRNAs. These similar effects may indicate that the RISC complexes present with the embryo have become saturated with siRNAs [53] or that p66Shc abundance, below a certain threshold, produces a limited amount of benefit on development that is not increased by further knockdown. A titration series of a single p66Shc siRNA molecule assessing developmental outcomes at different time points could establish the limits of such a threshold and any altered affects on embryo metabolism.

To further establish this relationship between improved developmental outcomes with reduced intracellular ROS levels and diminished DNA damage, RNAi-medicated knockdown of p66Shc was combined with moderate H_2_O_2_ treatments to determine whether p66Shc knockdown could confer stress resistance against exogenous ROS exposure. In our hands, p66Shc knockdown ameliorated ROS-induced p66Shc upregulation and activation reducing intracellular ROS generation and subsequent DNA damage in early embryos. These novel results in early embryos are analogous to previously published data on p66Shc deficient somatic cells that showed a widespread stress-resistent protective effect by reducing oxidative stress-induced cellular damage and apoptosis that prolonged lifespan in knockout mice [30], [36], [40]–[42]. Our initial H_2_O_2_ dose level of 100 µM was found to be excessive with virtually all embryos from each treatment groups succumbing to developmental failure. However, a second, lower dose level of 50 µM proved to be more amenable to improvement by p66Shc knockdown. This data confirms that while certain threshold levels of exogenous ROS treatment may be insurmountable, p66Shc knockdown is indeed able to confer protection against ROS-induced 2–4 cell permanent embryo arrest and apoptosis that rescues development to the blastocyst stage. As we have shown before [29], oxidant/stress treatment of embryos induces a massive upregulation of both p66Shc mRNA and protein levels. P66Shc expression and protein stability are highly responsive to intracellular ROS levels [54], [55] and are due, in part, to a ROS-mediated block in p66Shc ubiquitination and degradation throught Ser-54 and Thr-386 phosphorylation of p66Shc in a p38-dependent manner [56]. Enhanced stability of p53 mRNA transcripts has been induced by oxidative stress through a TIP 30-mediated process that triggers apoptosis [57]. Low levels of transcription has been observed before the major activation of genome transcription in early embryos [58], making early transcriptional/post-trancriptional regulation of p66Shc a possibility as well. Future studies thoroughly examining p66Shc’s transcriptional, post-transcriptional, translational and post-translational regulation will further delineate the molecular control and function of p66Shc expression during early embryo development.

P66Shc has been directly linked to ROS-induced apoptosis in cultured somatic cells by targeted mutation of p66Shc that rendered cells insensitive to p53-mediated apoptosis [36], [41], [59]. Oxidative stress promotes mitochondria translocation of p66Shc where it mediates H_2_O_2_/ROS production through its interactions and electron transfer with cytochrome c [38], either through activated PKC-β, which triggers Ser-36 phosphorylation of p66Shc [60]–[62] or through disulfide bond formation [63]. Thus, activated p66Shc is necessary for the stress-activated, p53-mediated release of cytochrome c from the mitochondrial membrane that triggers apoptosis [36], [41], [64]. Our results clearly demonstrate that activated p66Shc generates intracellular ROS triggering oxidative stress-mediated permanent cell cycle arrest and apoptosis in early bovine embryos. Preliminary data suggest that telomere uncapping is behind this DNA damage response (Betts and Madan, unpublished results). Interestingly, the decrease in apoptosis induced by p66Shc knockdown was substantially greater than the reduction in permanent 2–4 cell embryo arrest, possibly suggesting differential regulation for p66Shc functions at different stages of early development. Although Ser-36 phosphorylation of p66Shc has been widely implicated in its mitochondrial ROS regulatory function [36], [65], the forkhead transciption factor FOXO3a, which induces expression of key antioxidant enzymes such as MnSOD and catalase [66], is repressed by activated p66Shc through Akt-mediated phosphorylation, which results in FOXO3a exclusion from the nucleus [37], [67]. FOXO3a activity as well as catalase and MnSOD expression, has been found to be increased in p66Shc^−/−^ cells [37], [39], [42], [68]. We found that p66Shc knockdown significantly diminished the stress-induced nuclear exclusion of FOXO3a, increasing MnSOD levels and reducing superoxide generation in 5–8 cell embryos. Conversely, catalase/MnSOD mRNA and protein levels, when measured at the 2–4 cell stage, were virtually unaffected by stress treatments [29] suggesting that these antioxidants are not yet subject to transcriptional control by FOXO at this early cleavage stage. This discrepancy may account for the differential magnitude of early and late cleavage stage p66Shc knockdown embryos in their response to oxidative stress and developmental potential. A more indepth examination of the p53-p66shc-MnSOD network [55], [69] and pharmacological treatment of early embryos with inhibitors of p66Shc activation (e.g. PKC-β inhibitor hispidin) and its mitochondrial translocation (e.g. Src tyrosine kinase inhibitor PP2) will help delineate these varying functions during preimplantation development.

The ablation of p66Shc has been associated with a wide array of health benefits [39], [64], [70]. At the cellular level, these effects include an increased tolerance to oxidative stress-induced apoptosis [36], decreased generation of intracellular ROS [52], and increased antioxidant expression [67]. Collectively, these effects manifest at the organismal level in a variety of advantages including resistance to atherosclerosis [40], resistance to injury following ischemic reperfusion [71], and a significantly increased lifespan [36]. Our study has uniquely demonstrated that RNAi-mediated knockdown of p66Shc improves early embryo development by providing oxidative-stress resistance against permanent embryo arrest and apoptosis by diminishing intracellular ROS generation and increasing the antioxidant capabilities of early embryos. These mechanistic results indicate that the many benefits of p66Shc reduction previously cited within the literature also extend to improving the developmental competence of *in vitro* produced mammalian embryos. Future systems biology approaches focussed on redox signaling and metabolic networks will greatly enhance our understanding of the molecular and signaling processes governing preimplantation embryo development [72]. This new holistic information will enhance our ability to improve embryo culture conditions, to select the most competent embryos to transfer [73] and to minimize any detrimental effects of artificial microenvironments to maximize healthy postnatal development of *in vitro* produced livestock and individuals produced using assisted reproductive technologies [74].

## Materials and Methods

### *In vitro* Embryo Production

*In vitro* bovine embryo production was carried out as previously described [29]. Briefly, ovaries were freshly collected from a local abattoir (Better Beef Ltd, Guelph, Ont., Canada) into a pre-warmed (38.5°C) 0.9% saline solution. Upon laboratory arrival the ovaries were placed into sealed containers with fresh saline solution and maintained at 37.8°C in a humidified incubator until follicular aspiration (∼1–2 hr later). A vacuum pump (PipetAid LR68701) attached to an 18-gauge needle was used to aspirate cumulus-oocyte complexes (COCs) from 2–6 mm diameter follicles. COCs were collected into HEPES-buffered Ham’s F10 (Sigma-Aldrich Canada, Canada) and separated from follicular fluid by sedimentation (∼15 min.) COCs of appropriate morphology (spheroid, uniform opacity) were sorted into TCM-199 media (Sigma-Aldrich Canada, Canada) supplemented with 2% bovine serum (Cansera International, Canada), 0.2 mol/L sodium pyruvate (Sigma-Aldrich Canada, Canada), 0.2 mol/L L-glutamine (Sigma-Aldrich Canada, Canada), and 0.6% penicillin/streptomycin (Invitrogen, Canada). The final wash of TCM-199 was supplemented with 0.1 mg/ml of FSH, 1 mg/ml of LH and 1 mg/ml of estradiol (NIH, USA). COCs were matured in 80 µl drops (20 COCs/drop) of TCM-199 with hormone supplement under silicon oil (Paisley Products, Canada) for 22 hr at 38.5°C in a humidified 5% CO_2_ atmosphere. Matured oocytes were washed twice in HEPES-buffered Tyrode’s albumin-lactate-pyruvate medium (Sperm TALP) and twice in HEPES/Sperm TALP, supplemented with 20 mg/ml heparin (IVF TALP). Oocytes of appropriate morphology were sorted into oil-immersed 80 µl drops of IVF TALP (∼30/drop). Frozen-thawed bovine semen (Gencor, Guelph, Canada) was prepared by sperm “swim-up” in Sperm TALP for 1 hr at 38.5°C in a humidified 5% CO_2_ atmosphere [75]. The upper phase of the sperm/Sperm TALP solution was centrifuged at 300×g for 5 min to concentrate sperm in solution. COCs were co-incubated with sperm at a final concentration of approximately 1.0×10^6^ sperm/ml for 18 hr at 38.5°C in a humidified 5% CO_2_ atmosphere. Fertilization was concluded by gently vortexing oocytes for 3 min to remove remaining sperm and cumulus cells. Denuded presumptive zygotes were washed three times in EmbryoMax® (Millipore, USA) synthetic oviductal fluid (SOF) supplemented with 8.0 mg/ml BSA, 20 ml/L MEM non-essential amino acids, 10 ml/L BME essential amino acids, 0.4 mM sodium pyruvate and 500 U/ml Gentamicin. Presumptive zygotes were sorted into oil-immersed 30 µl drops (25 embryos/drop) of supplemented SOF and incubated in a Sanyo MCO-175M Tri-gas incubator at 38.5°C in a humidified 90% N_2_, 5% CO_2_, 5% O_2_ atmosphere for up to 8 days (192 hpi) or until further analysis was required.

### RNA Interference-mediated Knockdown of p66Shc

The p66Shc specific region (CH2) of the *SHC1* gene was queried for potential RNAi target sequences using the BLOCK-it™ RNAi designer software available on the Life Technologies™ website (http://rnaidesigner.invitrogen.com/rnaiexpress/). Potential siRNA molecules were additionally screened based on criteria established for rational siRNA design [43]. Two non-overlapping siRNA molecules (Supplemental Table S4), which best met the design criteria were synthesized (Life Technologies™, USA) and utilized on zygote-stage bovine embryos via microinjection. All p66Shc RNAi experiments were controlled with groups of non-injected embryos as well as embryos injected with a commercially available (Life Technologies, USA) medium G/C content negative control siRNAs. These non-injected and negative control siRNA-injected embryo groups were subjected to the same culture conditions and treatments as the p66Shc-specific siRNA-injected embryo groups (see below). The efficacy of the two p66Shc-specific siRNA molecules (RNAi-A and RNAi-E) were compared in terms of their respective abilities to knock-down p66Shc mRNA and protein. A titration series (0, 0.005 µM, 0.05 µM, 0.5 µM, and 5.0 µM) of the siRNA molecule determined to be most effective (RNAi-E) was injected into groups (n = 50/r, r = 3) of embryos which were analyzed for p66Shc mRNA content at 32 hpi. The longevity of the p66*Shc* knock-down effect over time was evaluated by observing the p66Shc mRNA content of RNAi-E injected groups (n = 50/r, r = 3) embryos at the 2–4 cell, 9–16 cell and blastocyst stages.

For RNAi-mediated knockdown experiments, siRNAs were introduced into zygote stage embryos via microinjection using a Leica® DMIRE2 inverted microscope equipped with a FemtoJet™ Injection System (Eppendorf International, USA). Holding pipettes were custom forged from Borosilicate capillaries (Sutter Instrument Company, USA) of an outer diameter of 1.0 mm, inner diameter of 0.78 mm using a Flaming/Brown Micropipette Puller (Sutter Instrument Company, USA) and a MF-900 Microforge (Narashige Group, Japan). Femtotip II (Eppendorf International, USA) injection pipettes of an outer 1063 diameter of 1.0 µM, inner diameter 0.5 µM, back-loaded with 1.5 µL of siRNA solution using Microloader® pipette tips (Eppendorf International, USA) were used as injection pipettes. Both injection and holding pipettes were controlled during microinjection using TransferMan NK2 micro-manipulators (Eppendorf International, USA), the pressure of the holding pipette being controlled via CellTram® vario manual injector (Eppendorf International, USA). Final injection volume was calculated using the following equation based on the manufacture’s instructions of the PLI-100 Pico-Injector (Harvard Apparatus Inc. USA): Volume (nL) = 0.17952× (tip inner diameter in µm)^3^× (Pressure in psi) × (Time in sec). Presumptive zygotes were collected at 8 hpi, vortexed for 3 min to remove excess sperm and cumulus cells, washed twice in pre-warmed Sperm TALP and transferred into 40 µL oil-immersed drops of Sperm-TALP in groups of 50. Embryos awaiting microinjection were maintained in a humidified incubator at 38.5°C with 90% N_2_, 5% O_2_, 5% CO_2_. Zygotes were divided into three groups (n ≥100): untreated embryos, embryos injected with a negative control siRNA molecule, and embryos injected with one of the p66Shc-specific siRNA molecules. A constant pressure of 15 hPA was present to prevent the backflow of medium into the injection pipette. An injection pressure of 75 hPA for 0.1 s was calculated to inject ∼ 10 pL of solution into each embryo per injection. Following microinjection, embryos were immediately transferred into SOF media and cultured as previously described [30]. Injection volume was optimized by treating groups (n = 50, r = 3) of embryos with a titration (10 pL, 50 pL, 75 pL) of injection volumes (of H_2_O) and assessing the rate of successful cleavage at 32 hpi. The efficiency of microinjection using the established optimal volume was evaluated by injecting groups (n = 75/r, r = 3) of embryos with a FITC-conjugated oligonucleotide and visually assessing the presence of the FITC-oligonucleotides under fluorescence microscopy (Supplemental Figure 1).

### Hydrogen Peroxide (H_2_O_2_) Treatments

Physiological levels of hydrogen peroxide (H_2_O_2_) treatment were employed as a source of exogenous ROS treatment because of its long physiological half-life (8 hr –20 days) and its ability to activate p66Shc [27], [36], [76], [77]. Embryos (n = 50, r = 3) were treated (and non-treated) at various developmental stages (0 hpi, 32 hpi, 48 hpi, 72 hpi) with exogenous H_2_O_2_ (50 µM or 100 µM)_._ In all cases, embryos were transferred into a Nunclon 4-well dish (Nalge Nunc International) containing 1.0 ml of H_2_O_2_-supplemented media and incubated at 38.5°C in a humidified 5% CO_2_/5% O_2_/90% N_2_ atmosphere for 1 hr. Treated embryos were then washed twice with and re-suspended in oil-immersed drops of or SOF medium. Developmental progression was assessed on day 8 (192 hpi). Group size, number of replications, and the timing of specific quantitative assays (DCF, Real Time PCR, etc.) are detailed within the results of each experiment.

### Total RNA Extraction

Total RNA was extracted from embryos using a combination of the QiaShredder™ and RNeasy® Micro Kits (Qiagen Inc., USA), according to the manufacturer’s instructions. Groups (n ^3^50) of embryos were pooled and homogenized with QiaShredder™ columns along with 20 ng of poly-A carrier RNA to facilitate maximum RNA yield. Homogenized samples were then passed through RNeasy® Micro nucleotide-binding membrane columns. Following several washes, DNA was eliminated from the columns via incubation with DNase I for 15 min at room temperature. Membrane bound RNA was then washed twice and eluted in 14 µL of RNase-free water. The integrity of total RNA was analyzed via 2% agarose gel electrophoresis. Spectrophometric measurement of the absorbance at 260 nm and the A260/A280 ratio assessed the purity and yield of total RNA respectively. RNA samples were used immediately for reverse transcription polymerase chain reaction (RT-PCR) or snap frozen in liquid N_2_ and stored at −80°C for future processing.

### Reverse Transcription Polymerase Chain Reaction (RT-PCR)

Reverse transcription polymerase chain reaction (RT-PCR) was performed using the Thermoscript RT-PCR system (Invitrogen™, USA) according to the manufacturer’s instructions. Eleven µL of total RNA was combined with 1 µL of 50 µM oligo(dT)20, and 1 µL of 10 mM dNTP mix. The samples were then denatured by incubation at 65°C for 5 min and placed on ice, after which the remainder of the cDNA reaction mixture was added: 4 µL of 5X cDNA synthesis buffer, 1 µL of 0.1M DTT, 1 µL of RNase OUT (Life Technologies) and 1 µL of Thermoscript RT (15 U/µL). The cDNA synthesis reaction was performed on a PTC-200 Peltier Thermocycler (MJ Research Inc., USA) using an incubation at 55°C for 60 min and terminated by a short incubation at 85°C for 5 min. Template RNA was eliminated from the samples by incubation with 1 µL of E. Coli RNase H (2 U/µL) at 37°C for 20 min. Finally, RT-PCR products were purified using the Qiaquick® PCR Purification Kit (Qiagen, USA) according to the manufacturer’s instructions. Briefly, the PCR reaction mix was centrifuged through a DNA-binding column that was washed several times before the purified DNA was eluted in 30 µL of PCR-grade water (Sigma-Aldrich Canada, Canada).

Messenger (m)RNA quantification of genes of interest was accomplished by Real Time PCR utilizing primers and PCR conditions that were either obtained from previous studies [78] or of our novel design (p66Shc) [29]. The 66 kDa isoform - specific region (CH2) of the homo sapiens *SHC1* gene was aligned with sequenced fragments of the Bos taurus genome (accession # XM_613898) obtained from the Genbank website (http://www.ncbi.nlm.nih.gov/sites/entrez?db=nucleotide). Based on sequence alignment, an area exhibiting a high degree of homology was targeted for oligonucleotide primer design using Primer3 v.0.4.0 software (http://frodo.wi.mit.edu/primer3/; Supplemental Table S5). To optimize and confirm the specificity of novel oligonucleotide primers, PCR amplifications were carried out on a PTC-200 Peltier Thermocycler (MJ Research Inc., USA) utilizing various temperatures and reaction mixes (Supplemental Table S5). To each sample the following mixture was added: 10X PCR buffer, 1.5–3 mmol/L MgCl_2_, 2.5 mmol/L dNTP mix, 100 pmol of forward and reverse primers (Supplemental Table S5), 2.5 IU Ampli Taq Gold™ polymerase (Applied Biosystems, USA), and PCR-grade water to a final volume of 50 µL. Each product underwent the following amplification program: activation at 95°C for 10 min, 45 amplification cycles of 95°C for 30 sec, annealing temperature for 30 sec, elongation at 72°C for 30 sec. The cycles were followed by a final elongation step at 72°C for 10 min. Aliquots of PCR products were separated by electrophoresis on a 2% agarose gel. The band of the predicted size was excised and from the gel and purified using Qiaquick® Gel Extraction columns (Qiagen, USA). Aliquots of the extracted PCR product was run on a subsequent 2% agarose gel along with a low mass DNA ladder and quantified by comparative densitometry. The identity of the PCR product was confirmed by having approximately 20 ng of purified PCR product sequenced by a DNA Analyzer (Applied Biosystems, USA) at the University of Guelph Laboratory Services Division.

### Real Time (LightCycler®) PCR

The expressivity of genes of interest was quantified by real-time, LightCycler® polymerase chain reaction (LC-PCR) using a LightCycler® 1.5 (Roche Applied Science, USA) employing a Faststart SYBR green I reaction mix (Roche Applied Science, USA) within 20 µL LightCycler® glass capillaries. Before gene transcript quantification, reaction conditions were optimized by performing LC-PCR runs with and without cDNA template in order to identify the melting temperatures of potential primer-dimers as well as predicted products. Optimized LC-PCR reaction conditions are described (Supplementary Table S5). The amplification program was as follows: pre-incubation for FastStart polymerase activation at 95°C for 10 min, 45 amplification cycles of denaturation at 95°C (20°C/sec) for 10 sec, annealing (20°C/sec) for 10 sec, elongation at 72°C (5°C/sec) for 20 sec, fluorescent acquisition (5°C/sec) for 1 sec. Upon the completion of the last cycle a melting curve was generated by starting the fluorescence acquisition at 65°C and taking measurements every 0.1°C until 95°C. A standard curve for each target gene was generated using Purified PCR products quantified through comparative densitometry and divided into six serial dilutions ranging from 1×10^2^ pg to 1×10^−4^ pg of template DNA. Histone 2A (H2A) was amplified in parallel to genes of interest as an endogenous control for PCR reaction efficiency. Negative control reactions lacking template DNA were run in parallel as controls against the formation of primer-dimers.

### Whole Mount Immunofluorescence

Embryos were immuno-stained using fluorophore-conjugated antibodies for the purpose of protein localization and protein quantification by measuring immunofluorescent signal intensities. An antibody titration series was used to determine the optimum concentration of each primary and secondary antibody (data not shown). Antibodies, producers and optimal dilutions are described in Supplemental Table S6. Embryos were washed twice in PBS at 38.5°C for 5 min and transferred into Fixing Solution (PBS +4% Paraformaldehyde (PFA)) for a 1 hr incubation at room temperature. Embryos at this point were either processed immediately or transferred into Embryo Storage Buffer (PBS +1% PFA) for short-term storage within humidified chambers at 4°C for a period of no greater than two weeks. Following fixation and/or storage, embryos were washed three times in PBS at 38.5°C for 5 min before being blocked and permeabilized simultaneously by incubation in Embryo Blocking Buffer (PBS +5% normal donkey serum, 0.01% Triton X-100) for 1 hr at room temperature. Embryos were then washed in PBS at 38.5°C for 20 min before being incubated in appropriate primary antibody, under non-saturating conditions, diluted in Antibody Dilution Buffer/Wash (PBS +1% normal donkey serum +0.005% Triton X-100) at 4°C overnight in a humidified chamber. Embryos were washed three times in Antibody Dilution Buffer/Wash at 38.5°C for 20 min before incubation in the appropriate secondary antibody diluted in Antibody Dilution Buffer/Wash within a dark humidified chamber at 4°C overnight. Under reduced light, embryos were washed three times in Antibody Dilution Buffer/Wash at 38.5°C for 45 min, with DAPI (1 mg/mL; 1∶2000 dilution, Sigma-Alrich Canada, Canada) added to the first wash to stain for DNA. Embryos were mounted on FisherBrand® Glass Slides (Fischer Scientific, USA) in a drop of DAKO Antifade (DAKO, USA) covered with No. 1 coverslips (Fischer Scientific, USA) and sealed with clear nail polish. Slides were imaged using an Olympus Fluoview Laser Scanning Confocal system on an IX81 inverted microscope running Olympus Fluoview v.4.3 software. Where protein quantity was to be determined by immunofluorescent signal intensity, micrographs of the appropriate fluorescent channel were converted into greyscale images and inverted through the black/white axis. Scion Image v.4.03 software was used to quantify the mean density of the immunofluorescent signal intensity present in each of the inverted greyscale micrographs. The mean signal intensity of three negative control micrographs (without primary antibody incubation) was subtracted from the measured values of the treatment micrographs to eliminate background fluorescence. The adjusted relative signal intensity values, represented in pixels, were plotted graphically, error bars representing the standard error of the mean.

### Dichlorofluorescein (DCF) Staining

Embryo H_2_O_2_ levels were quantified using the fluorescent probe 6-carboxy-2,7-dichlorodihydrofluorescein diacetate (DCF) (Molecular Probes) [79]. A stock solution of 5 mM DCF dissolved in DMSO (Sigma-Aldrich Canada, Canada) was diluted in PBS to a working concentration of 5 µM. Embryos were washed twice in PBS and placed 1271 into a Nunclon 4-well dish (Nalge-Nunc, USA) containing 500 µL of 5 µM DCF. Embryos were incubated at 38.5°C in a dark, humidified 90% N_2_, 5% CO_2_, 5% O_2_ atmosphere for 30 min. Stained embryos were washed twice with fresh PBS, sorted into a 72-well Microwell Minitray (Nalge Nunc International, USA) containing 10 µL of PBS and imaged (excitation at 495 nm and emission at 520 nm) using an Olympus IX81 inverted microscope capable of live-cell imaging. Image Pro Plus 5.0 image analyzing software was used to quantify the fluorescent signal intensity. The mean signal intensity of three negative control micrographs (embryos fixed with 4% PFA prior to DCF staining) was subtracted from the measured values of the treatment micrographs to eliminate background DCF signal. The adjusted relative signal intensity values, represented in pixels, were plotted graphically, error bars representing the standard error of the mean.

### TUNEL and Propidium Iodide Labelling

The apoptotic status of embryos was assessed through a combination of terminal deoxynucleotidyl transfer-mediated dUTP nick-end labelling (TUNEL) and propidium iodide (PI) nuclear labelling as previously described [80]. TUNEL staining was achieved using the *In Situ* Cell Death Detection Kit (Roche Applied Science, USA). Embryos were collected and washed three times in PBS/PVA and fixed with 4% PFA in PBS for 1 hr at room temperature. Fixed embryos were permeabilized in 0.5% Triton X-100 (Biorad Laboratories Ltd, Canada) in PBS for 1 hr at room temperature. Permeabilized embryos were incubated in the TUNEL mixture (deoxynucleotidyl transferase enzyme and fluorescein-dUTP in a 1∶9 ratio) for 1 hr at 37°C in the dark. Positive control embryos were pre-incubated with 5 IU DNase I (Promega, USA) for 20 min at 37°C. Negative control embryos were incubated in fluorescein-dUTP in the absence of the transferase enzyme. Embryos were then washed twice in 0.5% Triton X-100 in PBS, once in PBS/PVA and once in RNase buffer (40 mM Tris, 10 mM NaCl, 6 mM MgCl2, pH 8.0) before incubation with 0.1 mg/ml of RNase (Sigma-Aldrich) for 1 hr at 37°C in the dark. Following two washes in RNase buffer, the nuclei were stained with 5 mg/ml PI for 45 min at 37°C in the dark. Lastly, embryos were washed twice in PBS-PVA and mounted on slides with a 0.2% agar PBS solution. Stained embryos were imaged using an Olympus BX61 epifluorescence microscope running MetaMorph™ image analysis software. Embryos were considered to be apoptotic if >50% of stained nuclei exhibited fragmented and condensed morphology by PI staining in addition to positive TUNEL staining [81].

### Statistical Analysis

After homogeneity of variance of each sample was confirmed, statistical analysis was performed using the post-hoc Tukey-Kramer test [82]. Data were represented as means ± SEM. Differences between means were considered statistically significant when P<0.05.

## Supporting Information

Figure S1 **Microinjection of FITC-labeled oligonucleotide probes into bovine zygotes.**
(DOCX)

Figure S2 **Real-time quantification of p66Shc mRNA levels at various developmental timepoints after siRNA injection of bovine zygotes.**
(DOCX)

Figure S3 **Quantification of Serine 36-phosphorylated-p66Shc protein following RNAi-mediated knockdown of p66Shc.**
(DOCX)

Figure S4 **Localization of FOXO3a protein by immunofluorescent confocal microscopy.**
(DOCX)

Figure S5 **Real-time PCR quantification of Catalase and MnSOD mRNA following RNAi-mediated knockdown of p66Shc.**
(DOCX)

Figure S6 **Quantification of Catalase protein levels following RNAi-mediated knockdown of p66Shc.**
(DOCX)

Table S1 **Effects of microinjection volume on bovine embryo cleavage frequencies.**
(DOCX)

Table S2 **Microinjection efficacy of optimized settings for siRNA delivery.**
(DOCX)

Table S3 **Summary of FOXO3a nuclear exclusion under various IVF culture conditions and treatments.**
(DOCX)

Table S4 **Summary of p66Shc short interfering (si)RNA sequence information.**
(DOCX)

Table S5 **Summary of oligonucleotide primers and PCR conditions utilized.**
(DOCX)

Table S6 **Summary information on the primary antibodies utilized in this study.**
(DOCX)
