# Supplementary material for: The p66Shc Adaptor Protein Controls Oxidative Stress Response in Early Bovine Embryos
Source: PLoS One. 2014 Jan 24;9(1):e86978. doi: 10.1371/journal.pone.0086978 (PMC3901717; doi:10.1371/journal.pone.0086978)
Supplement: Figure S1 — Microinjection of FITC-labeled oligonucleotide probes into bovine zygotes. (DOCX) [file pone.0086978.s001.docx]

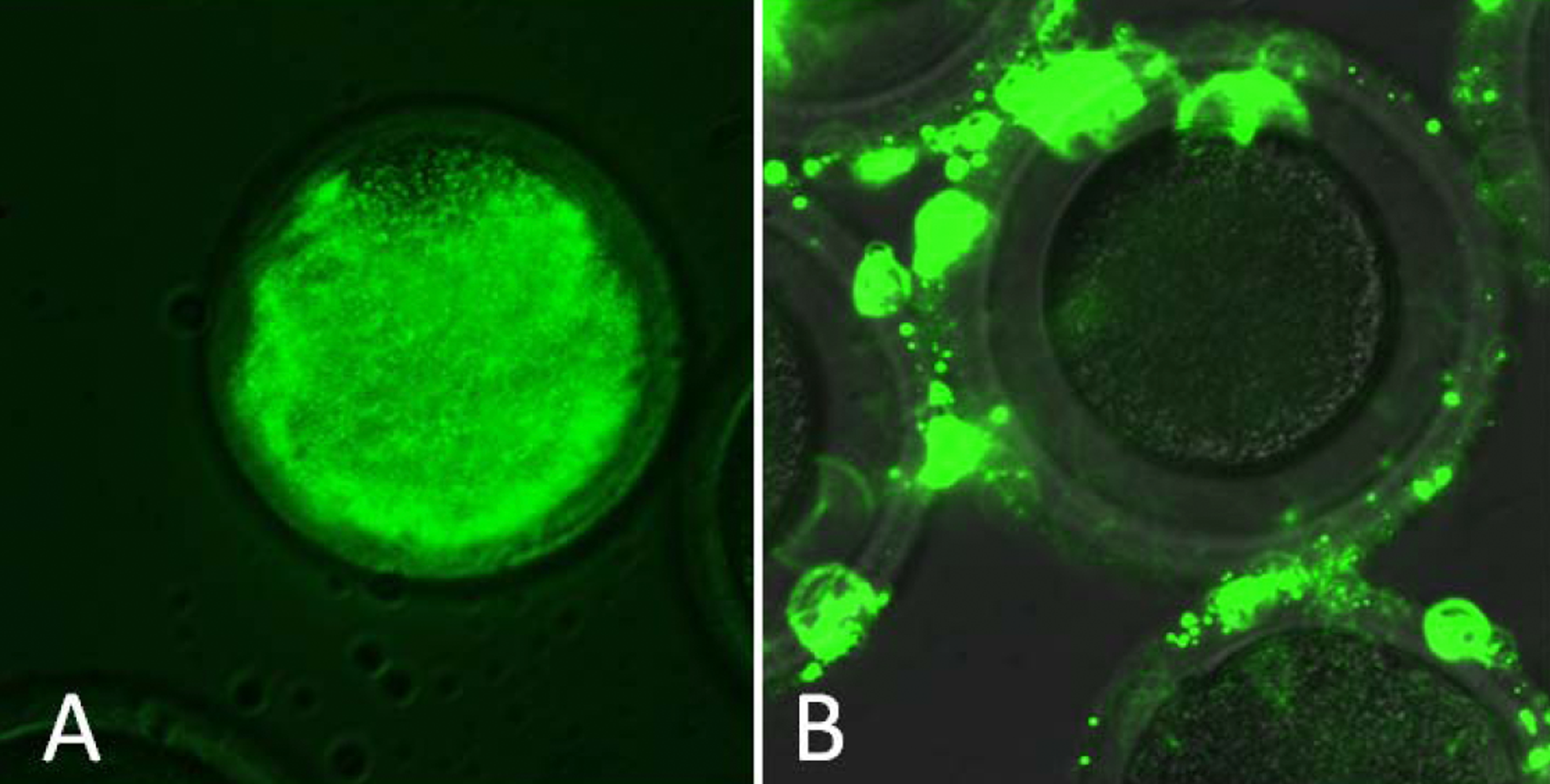


**Figure S1. Microinjection of FITC‐labeled oligonucleotide probes into bovine zygotes.** For determining injection efficiency and optimal injection volume, fluorescent-conjugated oligonucleotide probes were used to visualize the transfer of solution through the zona pellucida and cell membrane. (A) A representative zygote that has been successfully injected with FITC**‐**labeled oligonucleotides viewed under fluorescent illumination. (B) Attempts to use liposome-mediated transfer (Lipofectamine™ 2000) on bovine zygotes met with little success in penetrating through the embryo's zona pellucida.
