# Supplementary material for: The p66Shc Adaptor Protein Controls Oxidative Stress Response in Early Bovine Embryos
Source: PLoS One. 2014 Jan 24;9(1):e86978. doi: 10.1371/journal.pone.0086978 (PMC3901717; doi:10.1371/journal.pone.0086978)
Supplement: Figure S2 — Real-time quantification of p66Shc mRNA levels at various developmental timepoints after siRNA injection of bovine zygotes. (DOCX) [file pone.0086978.s002.docx]

**
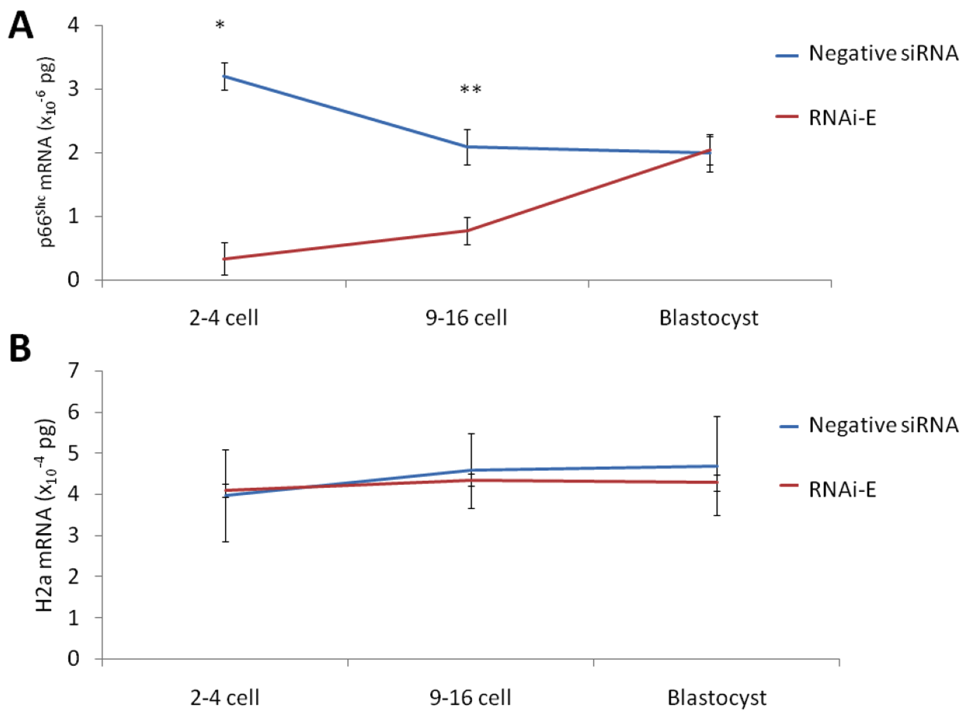
**

**Figure S2. Real-time quantification of p66Shc mRNA levels at various developmental timepoints after siRNA injection of bovine zygotes.** Total extracted RNA was pooled from groups of zygotes injected with either scrambled negative control siRNAs or p66Shc-specific RNAi‐E molecules. Embryos were harvested for total RNA at either the 2‐4 cell (32 hpi), 9‐16 cell (72 hpi) or the blastocyst stage (192 hpi). Asterisks (*) indicate significant differences (P<0.05). (A) P66Shc mRNA abundance was significantly decreased during the 2‐4 cell and to a lesser extent the 9‐16 cell stage. By the blastocyst stage of development, no significant difference was observed between RNAi injected and negative siRNA embryos. (B) Histone H2A mRNA was quantified in parallel as a control for siRNA specificity and PCR efficiency. No significant differences in H2A mRNA were noted between treatment groups. hpi = hours post insemination.
