# Supplementary material for: The p66Shc Adaptor Protein Controls Oxidative Stress Response in Early Bovine Embryos
Source: PLoS One. 2014 Jan 24;9(1):e86978. doi: 10.1371/journal.pone.0086978 (PMC3901717; doi:10.1371/journal.pone.0086978)
Supplement: Figure S3 — Quantification of Serine 36-phosphorylated-p66Shc protein following RNAi-mediated knockdown of p66Shc. (DOCX) [file pone.0086978.s003.docx]

**
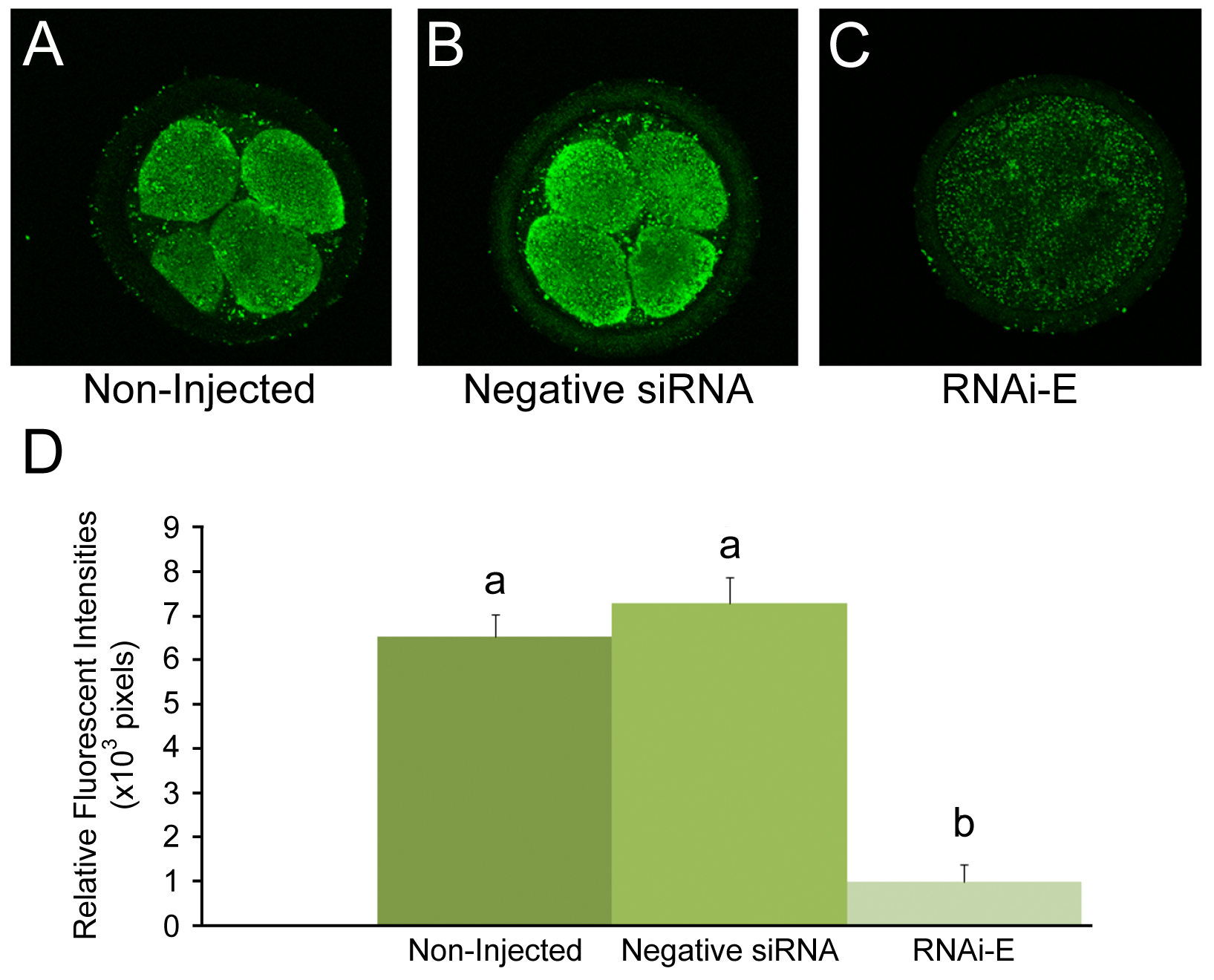
**

**Figure S3. Quantification of Serine 36‐phosphorylated‐p66Shc protein following RNAi-mediated knockdown of p66Shc.** Groups of 5‐8 cell embryos were immunostained for Serine 36‐phosphorylated‐p66Shc protein for quantification by relative immunofluorescent signal intensities. (A-C) Representative confocal images of S36-phosphorylated p66Shc immunostained 5-8 cell embryos that were either non‐injected (control), injected with negative control siRNA molecules, or injected with p66Shc-specific siRNA molecules (RNAi‐E). (D) Embryos injected with p66Shc-specific RNAi‐E molecules exhibited significantly lower levels of phosphorylated‐p66Shc protein than either control groups. Superscript letters denote significant differences (P < 0.05) in relative fluorescent intensities.
