# Supplementary material for: The p66Shc Adaptor Protein Controls Oxidative Stress Response in Early Bovine Embryos
Source: PLoS One. 2014 Jan 24;9(1):e86978. doi: 10.1371/journal.pone.0086978 (PMC3901717; doi:10.1371/journal.pone.0086978)
Supplement: Figure S4 — Localization of FOXO3a protein by immunofluorescent confocal microscopy. (DOCX) [file pone.0086978.s004.docx]

**
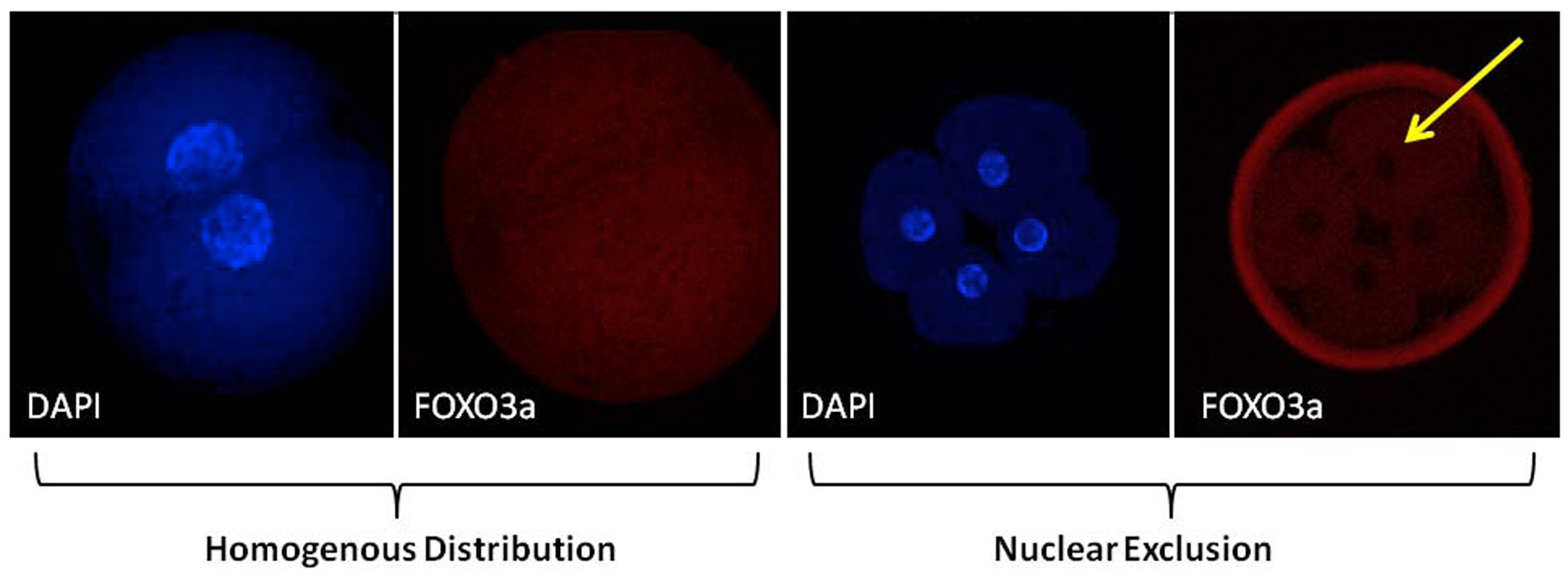
**

B

A

**Figure S4. Localization of FOXO3a protein by immunofluorescent confocal microscopy.**  Bovine embryos were co-stained with DAPI (nuclear stain, blue fluorescence) and anti-FOXO3a antibody (red fluorescence). (A) A representative 2-cell embryo exhibiting homogenous distribution of FOXO3a protein within the nucleus and cytoplasm. (B) A representative 4-cell embryo exhibiting nuclear exclusion pattern of FOXO3a staining (yellow arrow).
