# Supplementary material for: The p66Shc Adaptor Protein Controls Oxidative Stress Response in Early Bovine Embryos
Source: PLoS One. 2014 Jan 24;9(1):e86978. doi: 10.1371/journal.pone.0086978 (PMC3901717; doi:10.1371/journal.pone.0086978)
Supplement: Figure S5 — Real-time PCR quantification of Catalase and MnSOD mRNA following RNAi-mediated knockdown of p66Shc. (DOCX) [file pone.0086978.s005.docx]

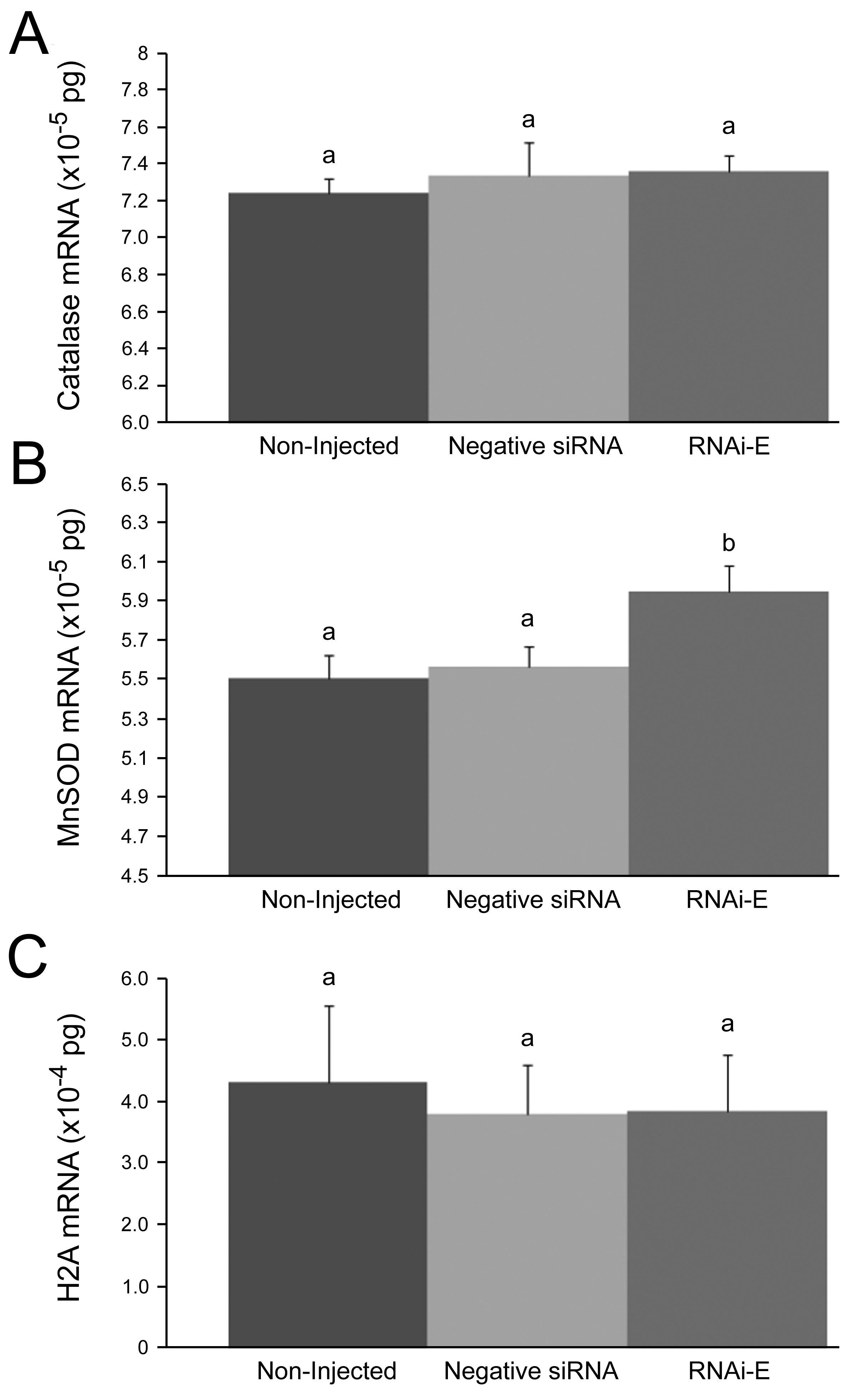


**Figure S5. Real‐time PCR quantification of Catalase and MnSOD mRNA following RNAi-mediated knockdown of p66Shc.** Total extracted RNA was pooled from groups 5‐8 cell stage embryos for the Real Time PCR quantification of Catalase and MnSOD mRNA. Embryos were: non‐injected (control), injected with negative siRNA, or injected with p66Shc siRNA molecule RNAi‐E. Significant differences (P < 0.05) are denoted by letters. (A) No significant differences in the abundance of catalase mRNA was observed between any of the treatment groups. (B) Embryos injected with RNAi‐E exhibited significantly higher levels of MnSOD mRNA than control groups. (C) H2A mRNA was quantified in parallel as a control of PCR efficiency. No significant differences in H2A mRNA content were noted between any groups treatment groups.
