# Supplementary material for: The p66Shc Adaptor Protein Controls Oxidative Stress Response in Early Bovine Embryos
Source: PLoS One. 2014 Jan 24;9(1):e86978. doi: 10.1371/journal.pone.0086978 (PMC3901717; doi:10.1371/journal.pone.0086978)
Supplement: Figure S6 — Quantification of Catalase protein levels following RNAi-mediated knockdown of p66Shc. (DOCX) [file pone.0086978.s006.docx]

**
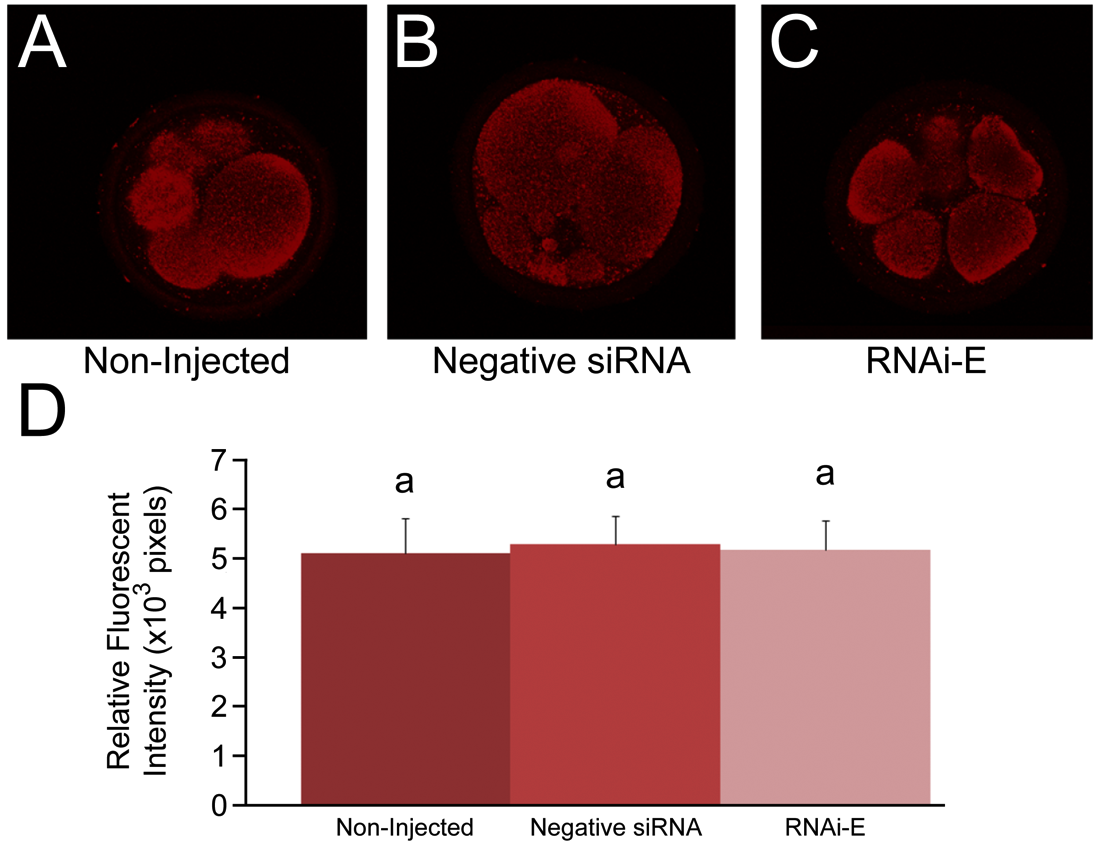
**

**Figure S6. Quantification of Catalase protein levels following RNAi-mediated knockdown of p66Shc.** Groups of 5‐8 cell embryos were stained for Catalase protein for quantification by relative immunofluorescent signal intensities. Representative confocal images of bovine embryos immunostained for catalase that were either (A) non‐injected (control), (B) injected with negative control siRNA molecule, or (C) injected with p66Shc-specific siRNA molecule RNAi‐E. Significant differences (P < 0.05) are denoted by letters. (D) No significant differences in relative fluorescent intensities were observed between any treatment groups.
