# Supplementary material for: The p66Shc Adaptor Protein Controls Oxidative Stress Response in Early Bovine Embryos
Source: PLoS One. 2014 Jan 24;9(1):e86978. doi: 10.1371/journal.pone.0086978 (PMC3901717; doi:10.1371/journal.pone.0086978)
Supplement: Table S1 — Effects of microinjection volume on bovine embryo cleavage frequencies. (DOCX) [file pone.0086978.s007.docx]

**Table S1.** Effects of microinjection volume on bovine embryo cleavage frequencies.

| **# Zygotes Injected** | **Injection Volume (pL)** | **% Embryo Cleavage (32hpi)** |
| --- | --- | --- |
| 50 (r=3) | 10 pL | 82.1 ± 3.2% |
| 50 (r=3) | 50 pL | 72.9 ± 2.9% |
| 50 (r=3) | 75 pL | 54.5 ± 4.1% |
