# Supplementary material for: The p66Shc Adaptor Protein Controls Oxidative Stress Response in Early Bovine Embryos
Source: PLoS One. 2014 Jan 24;9(1):e86978. doi: 10.1371/journal.pone.0086978 (PMC3901717; doi:10.1371/journal.pone.0086978)
Supplement: Table S2 — Microinjection efficacy of optimized settings for siRNA delivery. (DOCX) [file pone.0086978.s008.docx]

**Table S2.** Microinjection efficacy of optimized settings for siRNA delivery.

| **# of Zygotes Injected*** | **FITC-conjugated siRNA positive** | **Efficiency (%)** |
| --- | --- | --- |
| 75 | 72 | 96.0 |
| 75 | 68 | 90.7 |
| 75 | 75 | 100.0 |
| **225** | **215** | **95.3 ± 2.9%** |

* Injection pressure: 75 hPa; Constant pressure: 15 hPa; Injection time: 0.1 sec; Calculated injection volume: 10 pL
