# Supplementary material for: The p66Shc Adaptor Protein Controls Oxidative Stress Response in Early Bovine Embryos
Source: PLoS One. 2014 Jan 24;9(1):e86978. doi: 10.1371/journal.pone.0086978 (PMC3901717; doi:10.1371/journal.pone.0086978)
Supplement: Table S3 — Summary of FOXO3a nuclear exclusion under various IVF culture conditions and treatments. (DOCX) [file pone.0086978.s009.docx]

**Table S3.** Summary of FOXO3a nuclear exclusion under various IVF culture conditions and treatments.

| **Treatment^¥^** | **% Nuclei FOXO3a Excluded*** |
| --- | --- |
| 5% O_2_ (Control) | 4.6 ± 1.2^a^ |
| 20% O_2_ | 4.9 ± 1.6^a^ |
| Early Cleaving | 4.0 ± 0.9^a^ |
| Late Cleaving | 4.9 ± 1.3^a^ |
| 50 μM H_2_O_2_ | 54.4 ± 2.7^b^ |

^¥^n=50, 3 replicates; *Significant differences (P<0.05) are denoted by superscript letters
