# Supplementary material for: The p66Shc Adaptor Protein Controls Oxidative Stress Response in Early Bovine Embryos
Source: PLoS One. 2014 Jan 24;9(1):e86978. doi: 10.1371/journal.pone.0086978 (PMC3901717; doi:10.1371/journal.pone.0086978)
Supplement: Table S4 — Summary of p66Shc short interfering (si)RNA sequence information. (DOCX) [file pone.0086978.s010.docx]

**Table S4.** Summary of p66Shc short interfering (si)RNA sequence information.

| **Molecule** | **Target Sequence** | **siRNA Sense** | **siRNA Antisense** |
| --- | --- | --- | --- |
| **RNAi-A** | 5'-AATGAGTCTCTGTCATCGCTG-3' | 5'-UGAGUCUCUGUCAUCGCUGUU-3' | 5'-CAGCGAUGACAGAGACUCAUU-3' |
| **RNAi-E** | 5'-AGGGCAAATGAGGACGGGGA-3' | 5'-GGGCAAAUGAGGACGGGGAUU-3' | 5'-UCCCCGUCCUCAUUUGCCCUU-3' |
