# Supplementary material for: The p66Shc Adaptor Protein Controls Oxidative Stress Response in Early Bovine Embryos
Source: PLoS One. 2014 Jan 24;9(1):e86978. doi: 10.1371/journal.pone.0086978 (PMC3901717; doi:10.1371/journal.pone.0086978)
Supplement: Table S5 — Summary of oligonucleotide primers and PCR conditions utilized. (DOCX) [file pone.0086978.s011.docx]

**Table S5.** Summary of oligonucleotide primers and PCR conditions utilized.

| **Primer** | **Sequence (Forward, Reverse)** | **[MgCl_2_]**  **(mM)** | **Annealing Temp (^o^C)** | **Fluor. Acquisition Temp (^o^C)** | **Reference*** |
| --- | --- | --- | --- | --- | --- |
| **p66Shc** | 5'-GAGTCTCTGTCATCGCTGGA-3'  5'-CAGCTTCAGGCTGCTCATC-3' | 3.0 | 61 | 86 | Bain *et al*., 2013 |
| **Catalase** | 5'-GAACTGTCCCTACCGT-3'  5'-TCGTTGGCACTGTTGA-3' | 3.0 | 59 | 86 | Mathy-Hartert *et al*., 2008 |
| **MnSOD** | 5'-GCAAGTAAACCGTCAGC-3'  5'-AACTACCACCTCCTAGC-3' | 3.0 | 59 | 86 | Mathy-Hartert *et al*., 2008 |
| **H2A** | 5'-GTCGTGGCAAGCAAGGAG-3'  5'-GATCTCGGCCGTTAGGTACTC-3' | 3.0 | 55 | 88 | Robert *et al*., 2002 |

*Bain *et al.* (2013). *Mol Reprod Dev* **80**(1):22-34.;Robert *et al*. (2002). *Biol Reprod* **67**(5):1465-72.; Mathy-Hartert *et al.* (2008). *Osteoarthritis Cartilage* **16**(7):756-63.
