# Supplementary material for: The p66Shc Adaptor Protein Controls Oxidative Stress Response in Early Bovine Embryos
Source: PLoS One. 2014 Jan 24;9(1):e86978. doi: 10.1371/journal.pone.0086978 (PMC3901717; doi:10.1371/journal.pone.0086978)
Supplement: Table S6 — Summary information on the primary antibodies utilized in this study. (DOCX) [file pone.0086978.s012.docx]

**Table S6.** Summary information on the primary antibodies utilized in this study.

| **Antisera** | **Source (species antibody raised in)** | **Product Number (Abcam^®^ Inc., USA)** | **Dilution** |
| --- | --- | --- | --- |
| **p66Shc** | Rabbit | ab33770 | 1:100 |
| **Serine 36-phospho-p66Shc** | Mouse | ab54518 | 1:50 |
| **Gamma H2A.X** | Mouse | ab26350 | 1:100 |
| **FOXO3a** | Rabbit | ab12162 | 1:50 |
| **Catalase** | Rabbit | ab15834 | 1:50 |
| **MnSOD** | Rabbit | ab13534 | 1:50 |
